# Genetic assessment of eight zoo populations of golden snub‐nosed monkey (*Rhinopithecus roxellana*) implication to the conservation management of captive populations

**DOI:** 10.1111/eva.13726

**Published:** 2024-06-03

**Authors:** Jinxia Luo, Yansen Cai, Yuchen Xie, Xianlin Jin, Jianqiu Yu, Mei Xu, Xuanzhen Liu, Jing Li

**Affiliations:** ^1^ Key Laboratory of Bio‐Resources and Eco‐Environment (Ministry of Education), College of Life Sciences Sichuan University Chengdu China; ^2^ Department of Cell Biology and Genetic, School of Basic Medical Sciences Southwest Medical University Luzhou China; ^3^ Chengdu Zoo, Chengdu Research Institute of Wildlife Chengdu China

**Keywords:** captive population, genetic management, microsatellite, mitochondrial DNA D‐loop region, parentage identification, *Rhinopithecus roxellana*

## Abstract

Captive breeding programs play an important role in preserving the genetic diversity of endangered species. It is of utmost importance to conduct genetic assessment for captive populations in order to develop scientific breeding plans and conservation management strategies. Here, we genotyped 10 microsatellite loci and sequenced 368 bp of mitochondrial DNA control region for the golden snub‐nosed monkey (*Rhinopithecus roxellana*) from eight captive populations in China, and compared the genetic indices of captive populations with a wild population. Meanwhile, we performed paternity tests to verify the genealogical records and established genetic lineages. A total of 157 individuals were identified from 161 fecal samples, including 135 captive individuals (approximately 25% of captive individuals in China). Microsatellite analysis showed that the nine populations had moderate levels of genetic diversity, with polymorphism information content (PIC) ranging from 0.43 to 0.542; the genetic diversity of captive populations (average PIC: 0.503) was slightly higher than that of the wild population (PIC: 0.438). The Structure analysis indicated that individuals of the eight captive populations contained two different genetic components. We conducted either single‐blind or double‐blind paternity testing on 40 offspring of captive individuals and found that five offspring from two zoos (Nanjing Hongshan Forest Zoo and Shanghai Wild Animal Park) showed discrepant kinships from their pedigree records, probably due to the inaccuracies in pedigree records. By constructing genetic pedigrees, inbred offspring were found in Beijing Zoo, Shanghai Zoo, Hangzhou Zoo, and Chengdu Zoo. Analysis based on mitochondrial DNA showed a high level of genetic diversity in the eight captive populations (mean nucleotide diversity: 0.047). However, no nucleotide diversity was found in the wild population. This study conducted a genetic survey for captive golden snub‐nosed monkeys and will significantly benefit the genetic conservation management for captive populations in the future.

## 1 INTRODUCTION

According to the goals set by the United Nations Convention on Biological Diversity, comprehensive measures need to be taken globally to ensure the sustainability of biodiversity. Previous studies have primarily focused on the preservation of wild populations, with limited attention given to captive populations. Captive populations are equally important as they play a crucial role in preventing the extinction of endangered species and supplementing wild populations (Theodorou & Couvet, 2015). It is estimated that captive populations contribute to the preservation of approximately 15% of threatened vertebrates (Conde et al., 2011). However, factors such as small population size and unclear background make captive species more susceptible to inbreeding (Shan et al., 2014). Inbreeding leads to an increase in the frequency of homozygous genes, the accumulation of harmful mutations, and the loss of genetic diversity (Atkinson et al., 2018; Miller‐Butterworth et al., 2021). As a consequence of inbreeding, inbreeding depression can result in shorter average life expectancy and lower reproductive rates compared to wild populations (Frankham, 2005). To address these challenges, many strategies have been implemented by captive institutions, including the introduction of wild individuals, the use of assisted reproduction technologies to increase the reproductive output and the exchange of breeding individuals across different zoos (Apa & Wiechman, 2016; Lueders & Allen, 2020; Schmidt & Kappelhof, 2019). Since the 1990s, the Chinese Association of Zoological Gardens started to establish studbooks for captive animals using the Single Population Analysis & Records Keeping System (SPARKS), developed by the International Species Information System (ISIS). Information such as parentage, sex, transfers, breeding, diseases, and mortality of captive animals was carefully recorded (Liao et al., 2015). By analyzing studbook records, breeding plans have been developed that encourage collaboration among captive institutions and prioritize the selection of individuals with distant genetic relationships for breeding in order to prevent inbreeding (Wang et al., 2022). Despite of the manual studbooks, comprehensive genetic pedigrees based on molecular markers are still lacking for many captive populations in China, which will limit the scientific management of captive populations.

The golden snub‐nosed monkey (*Rhinopithecus roxellana*) is the flagship species in China and is listed as endangered by the IUCN (2021). Due to the changes in climate and terrain, as well as human activities, the habitat of *Rhinopithecus roxellana* has been fragmented and gradually divided into three subspecies: *R. r. roxellana* in Sichuan and Gansu Province, *R. r. qinlingensis* in Shaanxi Province and southern Qinling Mountains, and *R. r. hubeiensis* in Shennongjia, Hubei Province and its adjacent areas (Groves, 2001; Wang et al., 1998) (Figure S1). Since captive institutions began to breed and exhibit golden snub‐nosed monkeys in the 1960s, their number has grown rapidly (Yu et al., 2018). According to the 2019 International Studbook for Golden Snub‐nosed Monkey, the number of living captive golden snub‐nosed monkeys in China had reached 548, distributed in 49 captive institutions (Table S1). However, the studbook shows that the current captive individuals are descendants of a few founders captured from various regions such as Sichuan Province, Shaanxi Province, Gansu Province, and Hubei Province, which can further accelerate inbreeding and genetic diversity decreasing (Jangtarwan et al., 2020).

Although there have been studies on the genetic diversity of wild population (Kuang et al., 2019; Zhou et al., 2014), the research on the genetic evaluation of captive golden snub‐nosed monkeys is limited. Our previous research involving a single captive population is one of the few relevant studies (Cai et al., 2020). Given the continuing increase in captive golden snub‐nosed monkey number, a comprehensive genetic assessment, and accurate genetic pedigree is crucial for captive populations. Here, we combined 10 microsatellite loci and the hypervariable region I of the mitochondrial control region (HV I, mtDNA D‐loop region) sequences to assess the genetic diversity of eight captive (Beijing Zoo, BJZ; Beijing Wild Animal Park, BJW; Shaanxi Rare Wildlife Rescue Base, SXB; Nanjing Hongshan Forest Zoo, NJZ; Hangzhou Zoo, HZZ; Shanghai Wild Animal Park, SHW; Shanghai Zoo, SHZ; Chengdu Zoo, CDZ) and one wild (Pingwu, PW) golden snub‐nosed monkey populations. We also conducted paternity tests to verify the studbook records of these captive golden snub‐nosed monkeys. The objective of the study is to assess the genetic status of captive populations and to establish an accurate genetic pedigree, and this will be valuable for population genetic management and the healthy development of the captive golden monkey populations in the future.

## 2 MATERIALS AND METHODS

### 2.1 Sample collection and DNA extraction

We collected 114 fecal samples of golden snub‐nosed monkeys from 7 zoos, combined with 47 fecal samples previously collected (Cai et al., 2020) from Chengdu Zoo (CDZ) and a small wild population (PW) from Pingwu County, Sichuan Province (Table 1). Feces were obtained immediately after observing defecation behavior to ensure their freshness. We used disposable utensils and discarded the side that touched the ground to prevent potential cross‐contamination. With the help of the zookeepers, most of the samples could be accurately assigned to known individuals. However, for those housed in groups, it was difficult to observe defecation behavior individually, thus 14 fecal samples were randomly collected. All samples were separately stored in sterile bags and preserved at −80°C at the College of Life Sciences, Sichuan University. Genomic DNA was extracted using the TIANamp Stool DNA Kit (Tiangen Biotech, Beijing, China), following the manufacturer's instructions.

**TABLE 1:** Sample information of golden snub‐nosed monkeys.

Location	Living individuals in studbook	Sample size	Origin
Beijing Zoo (BJZ)	7	3	This study
Beijing Wild Animal Park (BJW)	26	24	This study
Shaanxi Rare Wildlife Rescue Base (SXB)	26	6	This study
Nanjing Hongshan Forest Zoo (NJZ)	26	6	This study
Hangzhou Zoo (HZZ)	9	9	This study
Shanghai Wild Animal Park (SHW)	58	49	This study
Shanghai Zoo (SHZ)	26	17	This study
Chengdu Zoo (CDZ)	16	25^a^	Cai et al. (2020)
Pingwu, Sichuan Province (PW)	–	22	Cai et al. (2020)
Total	194	161	

^a^ The number of living individuals was fewer than the sample size because these samples were from an earlier study (Cai et al., 2020) in which samples from dead individuals in the Chengdu Zoo were included.

### 2.2 Microsatellite amplification

The 14 microsatellite loci developed by Cai et al. (2020) were used for genetic evaluation. We excluded loci exhibiting either low sensitivity to low‐quality fecal samples or low polymorphism in the eight captive populations. We also excluded microsatellite loci located on the same chromosome to avoid potential linkage inheritance among loci (Li et al., 2002) by referring to the golden snub‐nosed monkey genome (Genbank Accession: NC_044551) (Wang et al., 2019). The chromosomal information of each locus is shown in the Table S2. Finally, only 10 microsatellite loci were selected for our genetic assessment. PCR was performed in a 20 μL volume including 10 μL Mix (2 × Rapid Taq Master Mix, P222‐AA, VAZYME BIOTECH, Nanjing), 0.5 μL each primer (25 μM solution), 2 μL template DNA (about 4 U) and 7 μL distillation‐distillation H_2_O (ddH_2_O). The amplification protocols were carried out as follows: 95°C predenaturation for 3 min, 95°C denaturation for 15 s, 51°C annealing for 15 s,72°C extension for 15 s, 35 cycles then 72°C final extensions for 3 min. For all PCR amplifications, we used the same amount of water and 10,000 times diluted amplification product as negative control and positive control, respectively, to reduce experimental errors. PCR products were detected using 1.5% polyacrylamide gel electrophoresis and then genotyped using ABI‐3730XL DNA Analyzer with GS‐500 Size as the standard internal reference by GeneMapper version 4.0 (Sangon Biotech, Shanghai). To obtain reliable genotypes, we considered a sample as heterozygous at a locus if the same heterozygous allele appeared at least twice and as homozygous if identical homozygous profiles were shown at least three times (Wultsch et al., 2014). Micro‐checker version 2.2.3 was used to detect technical scoring errors (Van Oosterhout et al., 2004).

### 2.3 Sex and individual identification

Primer pair: ZFX(F)5′‐TTATGGTGAAAGCCAAGAA‐3′; ZFY(R)5′‐GCAATTTCAGCAACATCTAAG‐3′ were amplified to determine the sex of golden snub‐nosed monkeys (Cai et al., 2020). The PCR system and amplification protocol were the same as that of microsatellites. PCR products were visualized on 1.5% polyacrylamide gel electrophoresis. The fragment sizes of X and Y chromosomes were 140 bp and 211 bp, respectively. Samples with two bands (the X and Y fragments) were identified as males, and one band (the X fragment) was identified as females.

The genotyping data of seven microsatellite loci (GSM47, GSM42, GSM04, GSM32, GSM21, GSM51, GSM75) and the results of sex identification were combined for individual identification (Cai et al., 2020) using Cervus version 3.0.7 (Kalinowski et al., 2007).

### 2.4 Data analysis based on microsatellite markers

Cervus was used to calculate allele number (A), observed heterozygosity (*H*_O_), expected heterozygosity (*H*_E_), and polymorphic information content (PIC). GENEPOP (https://genepop.curtin.edu.au/) was used to calculate the inbreeding coefficient (*F*_IS_); the pairwise fixation index (*F*_ST_) of golden snub‐nosed monkey populations was calculated by Arlequin version 3.5 (Excoffier & Lischer, 2010), 0 < *F*_ST_ <0.05 indicates little genetic differentiation; 0.05 < *F*_ST_ <0.15 indicates moderate genetic differentiation; 0.15 < *F*_ST_ <0.25 indicates large genetic differentiation; and *F*_ST_ >0.25 indicates very large genetic differentiation (Wright, 1978). STRUCTURE version 2.3.4 (Pritchard et al., 2000) was used for the genetic structure analysis. Ten independent runs were performed for each assumed number of cluster *K* from 1 to 10, and the optimal *K* was determined using the Delta K method (Evanno et al., 2005). POPGENE version 1.32 was used to calculate the Nei's genetic distances between populations (Nei, 1978). Cluster analysis was performed on standardized morphological data based on the Euclidian distance coefficient and un‐weighted pair group method with arithmetic means (UPGMA) using NTSYS‐pc version 2.10e (Rohlf, 1998).

We assigned parentage by using the pairwise‐likelihood method. Cervus were used to assign parents by calculating the LOD value (the logarithm of likelihood ratio) and Δ (the difference in LOD scores). The higher the LOD value, the more likely it is that the candidate parent will be the biological parent of the offspring; Δ represents the difference between the LOD value of the most likely parents and the second most likely parents (Marshall et al., 1998). The proportion of loci mistyped and error rate in likelihood calculations were all set as 1%; default values were used for confidence levels. To accommodate for experimental error and mutations, the possibility of biological parents can only be excluded when more than two loci are mismatched (Jones et al., 2010).

We conducted single‐blind paternity tests on 22 golden snub‐nosed monkey offspring who had only one parent in our collected samples. All adult males/females from the offspring's group were considered as candidate fathers or mothers, respectively. In addition, we conducted double‐blind paternity tests on 18 golden snub‐nosed monkey offspring whose recorded parents were both involved in the collected samples. All adult males and females in the offspring's group were added as candidate mothers and fathers, respectively. The genotypes of the recorded parents were then compared with other candidates to verify the lineage record. To determine the identity of 14 individuals lacking identity information, parent pair analysis of either double‐blind or single‐blind was conducted, with all adult samples selected as candidate fathers and mothers.

The sample size of BJZ was very small (three individuals), and according to the records, BJZ had frequent individual exchanges with BJW. Then, we combined the two zoos for paternity analysis and pedigree construction. Paternity results for CDZ and PW were obtained from Cai et al. (2020). The genetic pedigrees of the eight golden snub‐nosed monkey populations were constructed based on the paternity test results and visualized using Pedigraph (Garbe & Da, 2004).

### 2.5 Mitochondrial DNA D‐loop region amplification and data analysis

The mtDNA D‐loop region was amplified using the primer pair GH (5′‐AACTGGCATTCTATTTAAACTAC‐3′) and GL (5′ATTGATTTCACGGAGGATGGT‐3′), which were originally designed for the black snub‐nosed monkey (*Rhinopithecus bieti*) (Liu et al., 2007). PCR amplifications were performed in 20 μL containing 10 μL Mix (2 × Rapid Taq Master Mix, P222‐AA, Vazyme Biotech, Nanjing, China), 0.5 μL each primer (25 μM solution), 1 μL template DNA (about 2 U) and 8 μL ddH_2_O. PCR protocol was the same as that used for microsatellite amplification. Three microliters of PCR products were run on 1.5% agarose gel to detect whether samples were successfully amplified.

The amplified products were sequenced by the Beijing Genomics institution. The sequence alignment was performed in Mega version 5.2 (Tamura et al., 2011) using Clustal W (Thompson et al., 1994). The haplotype number (*H*), haplotype diversity (*h*), and nucleotide diversity (*π*) were calculated using DnaSP version 5.0 (Librado & Rozas, 2009).

## 3 RESULTS

### 3.1 Sex and individual identification

Combining the sex identification results with the genotyping data of 7 microsatellite loci, a total of 157 individuals were identified from 161 fecal samples (Table 2), of which 76 were males and 81 were females. Eight fecal samples belonged to four individuals: BJW8 and BJW9, SHZ14 and SHZ18, SHW41 and SHW29, and SHW40 and SHW42 were the same individual, respectively. The 14 fecal samples (BJW8, SHZ13, SHZ15, SHZ16, SHZ18, SHW9, and SHW40–SHW47) that lacked identity information were from 12 individuals.

**TABLE 2:** Sex and individual identification of nine golden snub‐nosed monkey populations.

Population	Number of individuals	Male	Female	Sex ratio (M:F)
BJZ	3	2	1	2
BJW	23	10	13	0.8
SXB	6	3	3	1
NJZ	6	4	2	2
HZZ	9	4	5	0.8
SHW	47	24	23	1.04
SHZ	16	7	9	0.78
CDZ	25	13	12	1.08
PW	22	9	13	0.69
Total	157	76	81	0.94

### 3.2 Establishment of microsatellite dataset of captive golden snub‐nosed monkeys

**FIGURE 1:**
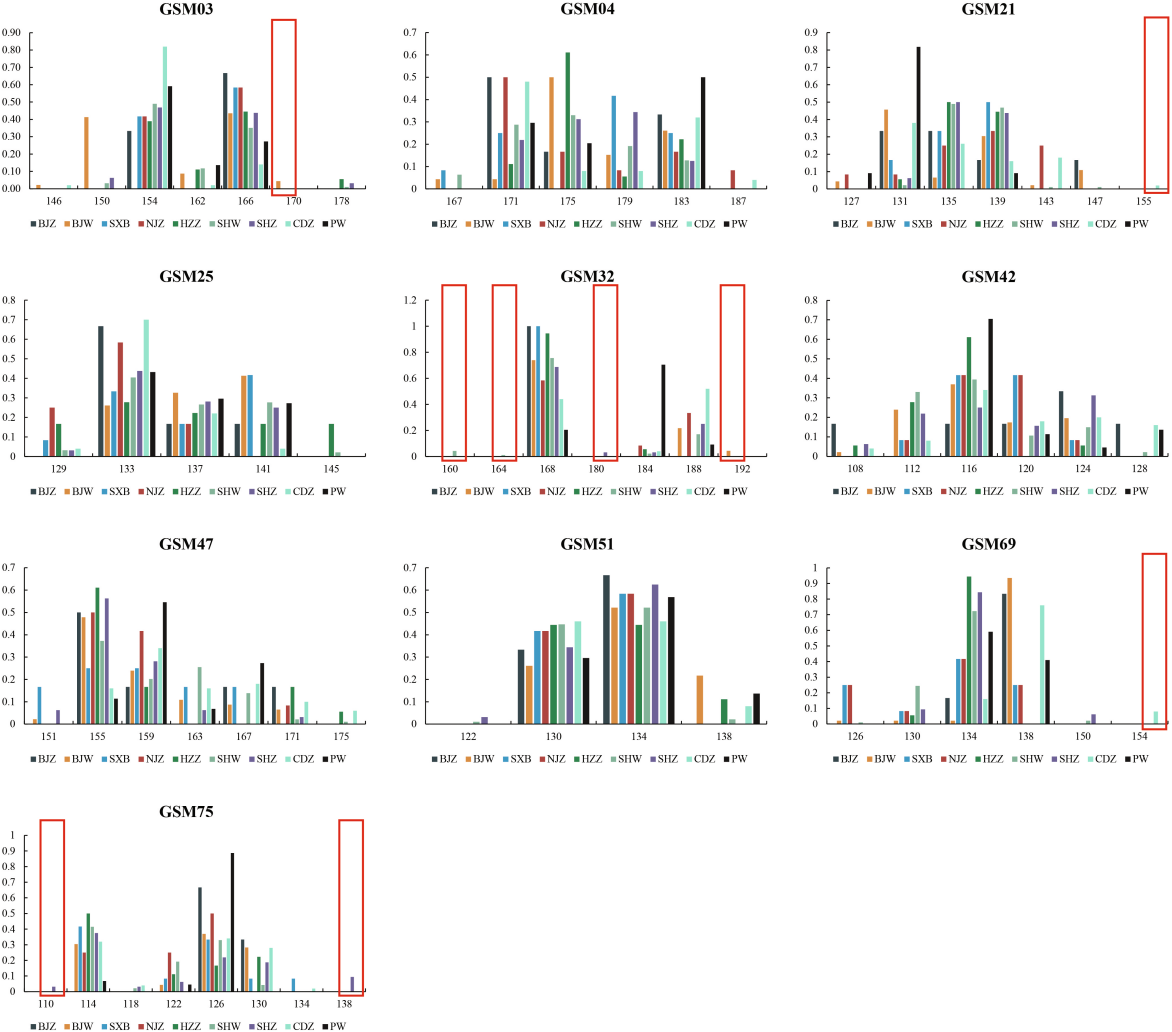
Allele size (*x*‐axis) and distribution frequency (*y*‐axis) of nine golden snub‐nosed monkey populations based on ten microsatellite loci, private alleles were framed by red boxes.

The genotyping data based on 10 microsatellite loci for each individual were organized into a microsatellite dataset of captive golden snub‐nosed monkeys (Table S3). Together with the genotyping data of PW wild population based on the same microsatellite loci, we found that most loci showed similar allele sizes and distribution frequencies among the nine populations, while several loci showed variation in the allele sizes and distribution frequencies (Figure 1). For example, the allele frequency of 184 bp in locus GSM32 in PW was over 70%, while in other populations, it was below 10%; the frequency of 150 bp in locus GSM03 in BJW was 41%, which was significantly higher than that in other populations (<6%). Private alleles were found in a few populations: two in BJW, two in SHW, three in SHZ, and two in CDZ (red boxes in Figure 1).

### 3.3 Genetic diversity based on microsatellite markers

A total of 63 alleles were detected at 10 loci in 157 individuals from nine golden snub‐nosed monkey populations (Table 3, Tables S4 and Table S5). SHW had the highest allele number (49) and BJZ had the lowest (28). The values of Ho ranged from 0.5 (BJZ and CDZ) to 0.85 (SXB), H_E_ ranged from 0.506 (PW) to 0.634 (SXB), and PIC ranged from 0.43 (BJZ) to 0.542 (SHW), respectively. PIC is an indicator of polymorphism in gene fragments (Botstein et al., 1980). Among the nine populations, BJZ, NJZ, HZZ, and PW showed moderate polymorphism (0.25 < PIC < 0.5); BJW, SXB, SHW, SHZ, and CDZ showed high polymorphism (PIC > 0.5). Positive mean inbreeding coefficient (*F*_IS_) values were found in BJZ, NJZ, and CDZ, along with lower observed heterozygosity (*H*_O_ < *H*_E_), indicating the possible occurrence of inbreeding in the captive populations.

**TABLE 3:** Genetic diversity of nine golden snub‐nosed monkey populations based on microsatellite loci.

Population	*A*	*N*	*H*_O_	*H*_E_	PIC	*F*_IS_
BJZ	28	3	0.5	0.587	0.43	0.178
BJW	44	23	0.748	0.601	0.536	−0.251
SXB	34	6	0.85	0.634	0.517	−0.389
NJZ	31	6	0.583	0.594	0.474	0.012
HZZ	35	9	0.634	0.54	0.458	−0.228
SHW	49	47	0.723	0.613	0.542	−0.183
SHZ	42	16	0.65	0.607	0.527	−0.074
CDZ	44	25	0.5	0.603	0.537	0.174
PW	31	22	0.559	0.506	0.437	−0.109
Total	63	157	0.652	0.665	0.606	–

*Note*: Allele number at the locus (*A*), number of individuals typed at the locus (N), observed heterozygosity (*H*_O_), expected heterozygosity (*H*_E_), polymorphic information content (PIC), inbreeding coefficient (*F*_IS_).

### 3.4 Genetic structure and differentiation of populations

**FIGURE 2:**
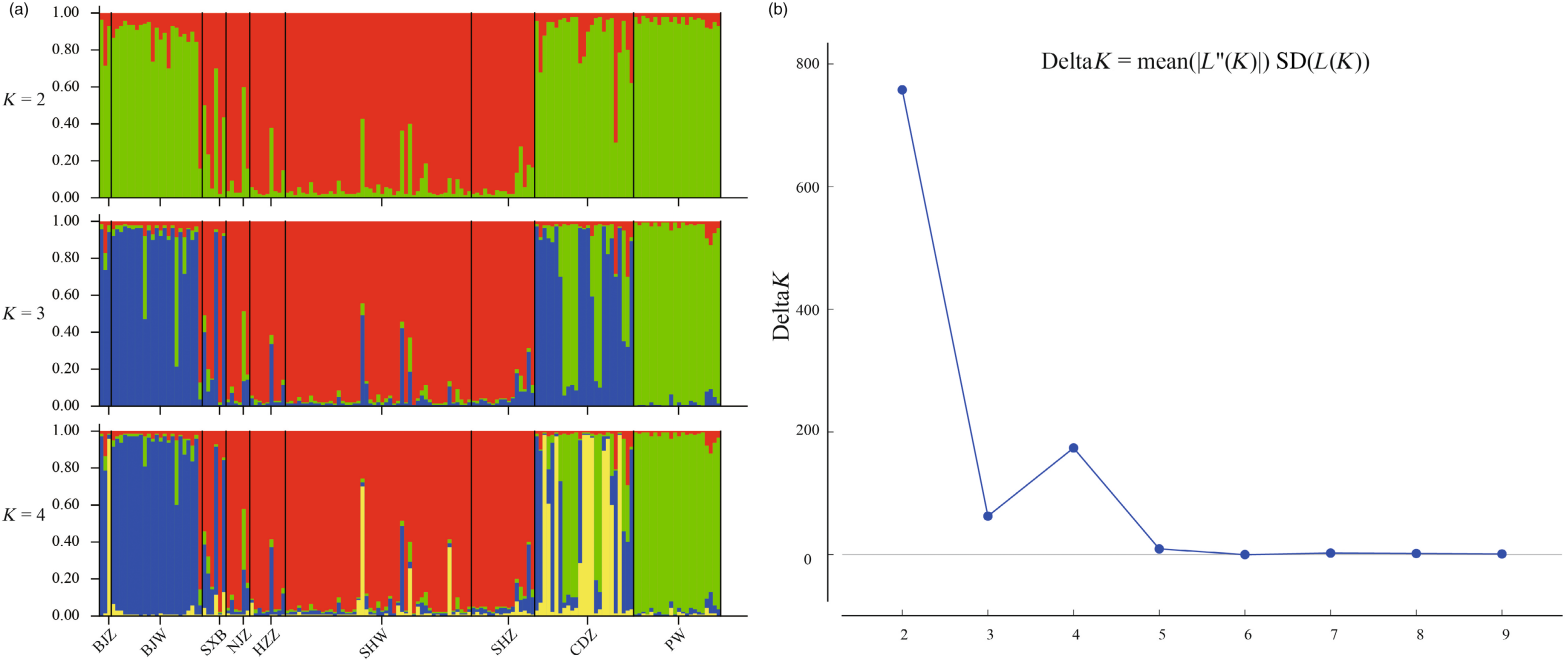
Population genetic structure analysis and *K* value changes. (a) Population genetic structure analysis, different colors represent different genetic components. (b) Variation curve of △*K* with *K* values. *K* = 2 is the optimal population allocation number.

Bayesian analysis implemented by the STRUCTURE identified *K* = 2 as the most likely number of clusters (high peak occurred at Delta *K* = 2) (Figure 2b), where the BJZ, BJW, CDZ, and PW grouped together as one cluster, SXB, NJZ, HZZ, SHZ, and SHW formed another cluster (Figure 2a). The two genetic components may represent two different subspecies. The green cluster included individuals from PW wild population, who was designated to be *R. r. roxellana* (Groves, 2001; Wang et al., 1998). Therefore, the genetic component of golden monkeys in BJZ, BJW, CDZ is more similar to *R. r. roxellana*. Whereas monkeys in other zoos (SXB, NJZ, HZZ, and SHZ) shared a genetic component (red) with two wild *R. r. qinlingensis*. However, because only five individuals were captured from the wild and no *R. r. hubeiensis* was included in our samples (Table S6), the exact subspecies of these captive individuals needed further validation. Notably, our structural analysis indicated that the genetic component of the captive individuals of the eight zoos was complex, likely due to a mixture of different subspecies.

When *K* = 3 and *K* = 4, the genetic component of PW remained simple; the primary genetic component of BJZ and BJW exhibited similarity, while NJZ, HZZ, SHZ, and SHW belonged to another main component. Compared to wild population, the genetic component of captive populations was quite complex. This was particularly severe in CDZ and SXB.

The analysis of population genetic differentiation (*F*_ST_) showed that there was a moderate degree of differentiation between the nine golden snub‐nosed monkey populations, with an average value of 0.116 (ranging from 0.009 to 0.264) (Table 4). The highest levels of genetic differentiation were detected between PW wild population and different captive populations (*F*_ST_ >0.15). The pairwise Nei's genetic distances among populations demonstrated a narrow range from 0.031 (SHW and SHZ) to 0.511 (HZZ and PW). The UPGMA cluster constructed based on microsatellite genotypes (Figure S2) indicates that the captive individuals did not cluster by captive institutions; individuals from BJZ, BJW, CDZ, and PW were more closely related, which is consistent with the Structure results.

**TABLE 4:** Genetic differentiation index (*F*_ST_) values (below the diagonal) and Nei's genetic distance (above the diagonal) in the nine golden snub‐nosed monkey populations based on microsatellite loci.

Population	BJZ	BJW	SXB	NJZ	HZZ	SHW	SHZ	CDZ	PW
BJZ	**–**	0.104	0.145	0.298	0.328	0.276	0.251	0.152	0.329
BJW	0.021**	**–**	0.199	0.438	0.325	0.310	0.309	0.198	0.351
SXB	0.027**	0.085**	**–**	0.209	0.161	0.083	0.099	0.279	0.398
NJZ	0.114**	0.181**	0.078**	**–**	0.134	0.093	0.119	0.303	0.376
HZZ	0.153**	0.160**	0.074**	0.062**	**–**	0.059	0.063	0.473	0.511
SHW	0.109**	0.143**	0.026**	0.035**	0.026**	**–**	0.031	0.316	0.426
SHZ	0.097**	0.141**	0.031**	0.046**	0.025**	0.009**	**–**	0.328	0.469
CDZ	0.049	0.099**	0.118**	0.134**	0.208**	0.145**	0.148**	**–**	0.277
PW	0.179**	0.189**	0.205**	0.204**	0.264**	0.208**	0.230**	0.159**	**–**

*Note*: ** Denotes significant difference after Bonferroni correction (*p* < 0.01).

### 3.5 Single‐blind paternity testing

A total of 226 parent pairs were analyzed on the 22 golden snub‐nosed monkey offspring by single‐blind paternity testing. The results showed that 21 out of the 22 monkey's recorded fathers/ mothers were supported to be the biological fathers/mothers in the paternity test (Table S7). Of these, 11 recorded fathers/mothers had the highest LOD score among all candidates. Other 10 recorded fathers/mothers were not supported by the highest LOD scores; however, after manual verification of breeding records, rearing cage records, consanguineous relatives, and age of individuals, and elimination of these confounding factors, the paternity testing results were consistent with their studbook records.

Only the paternity testing result of one individual, NJZ5 from Nanjing Hongshan Forest Zoo, was inconsistent with its studbook records. Four mismatched loci were found between NJZ5 and its recorded father (NJZ2), excluding the possibility of a biological relationship (Table 5). Furthermore, the remaining candidate fathers also exhibited over two mismatched loci with NJZ5. Given that only 6 out of 26 individuals from NJZ were sampled in our study, it is likely that samples of the biological father of NJZ5 were not collected in this study.

**TABLE 5:** Paternity tests on the five golden snub‐nosed monkeys exhibiting inconsistencies between the genetic identification results and the pedigree records.

Offspring ID	Parent/parents ID	Number of analyzed loci	Mismatched loci	LOD	Δ	Confidence
NJZ5 ♀	NJZ6 ♂	10	3	−9.95		
	*NJZ2 ♂*	10	4	−16.2		
SHW10 ♂	SHW46 ♀ & SHW08 ♂	10	3	−6.11		
	Other 6 combinations	10	3–5	−20 to −6.53		
	*SHW47 ♀ & SHW38 ♂*	10	5	−20.2		
SHW13 ♀	SHW47 ♀ & SHW25 ♂	10	0	5.15	0.7	>95%
	Other 179 combinations	10	0–5	−17 to 4.45		
	*SHW16 ♀ & SHW3 ♂*	10	5	−17		
SHW20 ♀	SHW44 ♀ & SHW32 ♂	10	2	−0.39		
	Other 97 combinations	10	2–5	−16.5 to −2.39		
	*SHW35 ♀ & SHW38 ♂*	10	4	−16.7		
SHW37 ♂	SHW18♀ & SHW21♂	10	1	1.56	1.56	>95%
	Other 118 combinations	10	2–5	−2.34 to −18.6		
	*SHW47♀ & SHW38♂*	10	5	−18.7		

*Note*: Pedigree‐recorded parent pairs were italics.

### 3.6 Double‐blind paternity testing

A total of 3487 parental pairs were analyzed on 18 golden snub‐nosed monkey offspring (Table S8). The results showed that 14 of the 18 offspring had parentages consistent with the genealogical records. Four of them had their parental pairs supported by the highest LOD value. While in the other 10 offspring, the highest LOD values were not from the recorded parents but from the candidates who were close relatives (full siblings, aunt, etc.) to the offspring. After excluding confounding factors from close relatives, the recorded parents could be supported by the biological fathers and mothers.

However, the results of the genetic identification of the remaining four individuals (SHW10, SHW13, SHW20, and SHW37) did not match their studbook records (Table 5). For example, all candidate parent pairs had at least three mismatched loci with SHW10, which means its biological parents were not included in this study. In the case of SHW13, although we identified parent pairs with high LOD values and high confidence, there were no mating records between the pairs according to the studbook; thus, we cannot confirm that these parent pairs were their biological parents.

### 3.7 Individual identification by paternity testing

For 12 individuals with unknown identity information, we identified studbook numbers for two individuals (BJW8, SHZ41) by individual identification, and paternity tests were conducted on the remaining ten individuals to determine their identity (Table 6). Firstly, the double‐blind paternity testing was applied to find candidate parent pairs of unknown individuals. The identity of them might be determined by combining with the studbook records. If the results of double‐blind paternity testing were not confident, the single‐blind paternity testing was then conducted to find individuals with the closest kinship to the unknown individual; thus, the identity might be determined. With the method and careful analysis, studbook numbers of eight unknown golden snub‐nosed monkeys were successfully determined, and only two individuals failed to be identified.

**TABLE 6:** Parentage identification of 10 golden snub‐nosed monkeys with unknown identities.

Offspring ID	Candidate parent (s)	Number of mismatched loci	LOD	Δ	Confidence	Identified studbook number
SHZ13 ♂	SHZ7 ♀ & SHZ5 ♂	3	−7.15			SHZ13 to be 829
	Other 39 parent pairs	4–6	−26.9 to −11.4			
	SHZ7 ♀	1	−0.769			
	SHZ8 ♀	1	−2.27			
SHZ15 (SHZ18) ♂	SHZ04 ♀ & SHZ18 ♂	3	−6.17			SHZ15 (SHZ18) to be 888
	Other 39 parent pairs	4–8	−34.1 to −11.8			
	SHZ4 ♀	2	−3.94			
SHZ16 ♀	SHZ2 ♀ & SHZ12 ♂	3	−5.65			SHZ16 to be 1095
	Other 39 parent pairs	3–7	−26.0 to −7.45			
	SHZ12 ♂	0	4.91	3.7	>95%	
	SHZ5 ♂	0	1.2			
SHW9 ♂	SHW46 ♀ & SHW31 ♂	1	3.05	3.05	>95%	Failed
	SHW46 ♀ & SHW3 ♂	2	−0.91			
	Other 206 parent pairs	2–8	−31.71 to −0.91			
SHW40 (SH®W42) ♂	SHW47 ♀ & SHW38 ♂	0	1.1			SHW40 (SHW42) to be 1047 SHW47 to be 677
	Other 236 combinations	1–8	−30.76 to 0.51			
SHW43 ♀	SHW35 ♀ & SHW38 ♂	2	−5.78			SHW43 to be 1098
	Other 215 combinations	3–8	−32.65 to −6.29			
SHW44 ♀	SHW46 ♀ & SHW26 ♂	1	2.48	2.48	>95%	Failed
	Other 220 combinations	2–8	−31.14 to −1.81			
SHW45 ♀	SHW6 ♀ & SHW23 ♂	1	0.55	0.28	>80%	Physical separation
	Other 8 combinations	1–3	−5.6 to 0.27			
	SHW39 ♀ & SHW38 ♂	2	−5.77			SHW45 name: 208
	Other 205 combinations	2–8	−30.87 to −4.94			
SHW46 ♀	SHW47 ♀ & SHW9 ♂	2	14	1.4		
	Other 220 combinations	2–8	−30.99 to −0.63			
SHW47 ♀	SHW46 ♀ & SHW23 ♂	0	7.59	5.13	>95%	Lacking mating records
	Other 214 combinations	1–8	−32.61 to −0.26			

To determine the studbook numbers of two unknown individuals (SHW46 and SHW47), we combined paternity testing results with reproduction records. Taking SHW46 as an example, the double‐blind paternity testing confirmed that SHW49 was the offspring of SHW46 & SHW38. Given that the studbook numbers of SHW49 and SHW38 were known, according to the reproduction records from zoo, the identity of SHW46 was confirmed to be studbook number 479 (ID: T3).

The identity information of three individuals (SHZ13, SHZ15, and SHZ16) was identified through single‐blind paternity testing. In the case of SHZ16 from Shanghai Zoo, all adult pairs in SHZ were selected as candidate parents for double‐blind identification. None of the parent pairs could be confirmed with high confidence. We further searched for single parents or individuals closely related to SHZ16 through single‐blind paternity testing, and two males (SHZ12 (born in 2015) and SHZ5 (born in 2013)) were supported with high LOD values (4.91 and 1.2, respectively) and matched at all 10 loci. Combined with breeding records, we found that Anna (studbook number: 1095, born in 2019) was a sibling and half‐sibling to SHZ12 and SHZ5, respectively. The studbook number of SHZ16 (1095) was finally identified.

As for the unknown individuals of SHW44 and SHW9, the double‐blind paternity testing identified parent pairs with high confidence (>90%) and only one mismatched locus. However, the reproduction records from zoos showed the identified parent pairs had never mated before. It is attributed to two possible reasons: either the candidate's parents were close relatives to the unknown individuals, or the manual reproduction records made some mistakes that often occurred in captive population management.

### 3.8 Pedigree construction

**FIGURE 3:**
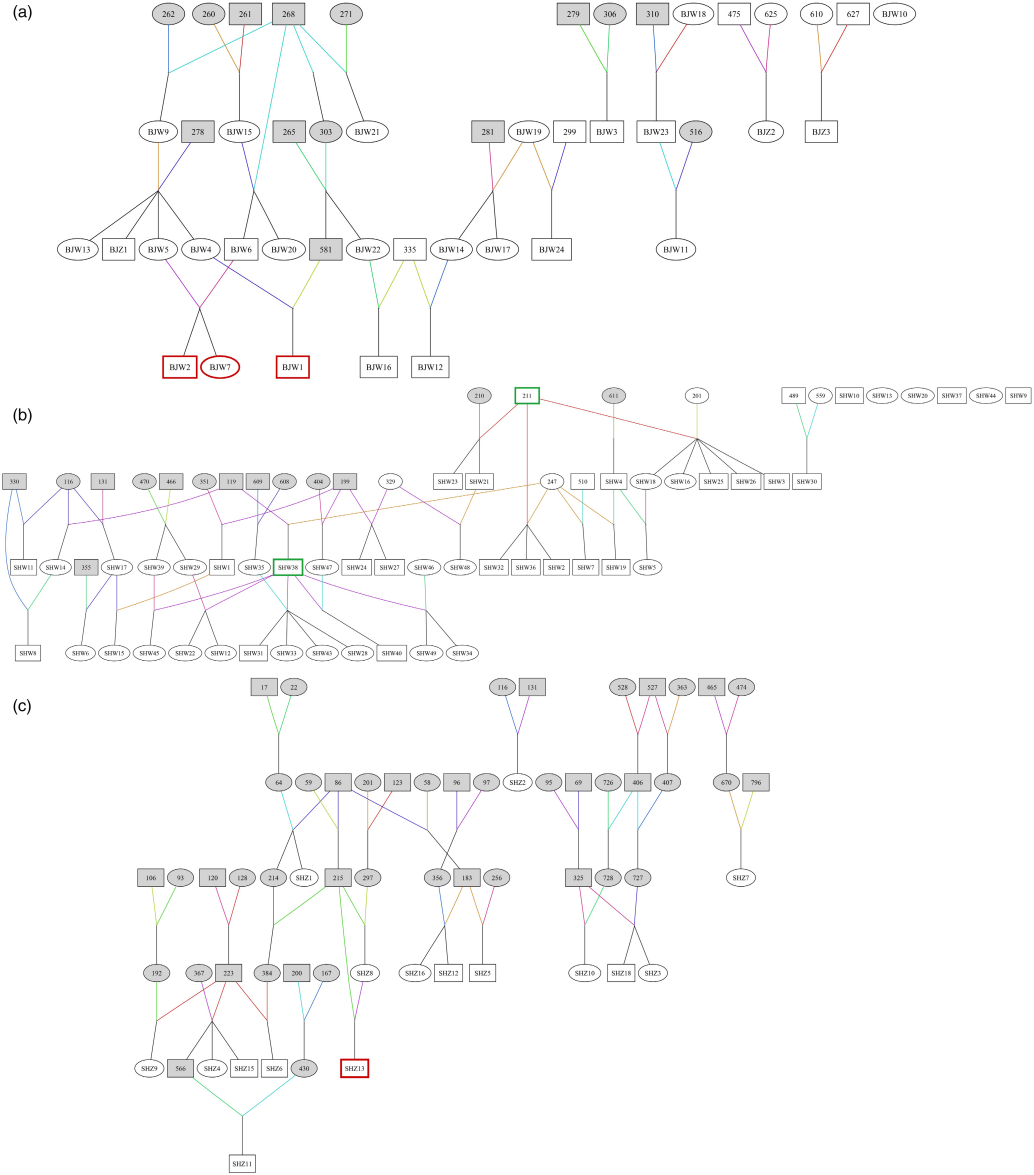
Genetic pedigree of three captive golden snub‐nosed monkey populations. □: male, ○: female, dead individuals were filled in gray; inbred individuals were marked in red. Various colored lines represent different mating combinations of parents. When two colored lines intersect, if the black solid line leads down from the intersection point, it represents the following individuals as their offspring. (a) Genetic pedigree of BJZ and BJW. (b) Genetic pedigree of SHW. (c) Genetic pedigree of SHZ.

Genetic pedigree was constructed for the eight captive golden snub‐nosed monkey populations based on the corrected parent–child relationships. Genetic pedigrees of BJZ & BJW, SHW, and SHZ were shown in Figure 3, while the remaining population pedigrees were shown in the Figures (S3–S6). Our results indicated that inbreeding occurred in some captive populations. We identified two offspring from mating between first‐order relatives and one offspring from mating between second‐order relatives in BJW (red boxes in Figure 3a). Additionally, one offspring (SHZ13) from mating between first‐order relatives in SHZ (red boxes in Figure 3c) were identified. Currently, no inbred individual was found in the samples from SXB (Figure S3), NJZ (Figure S4), and SHW; however, most of the juvenile golden monkeys in SHW were offspring of two males (211♂, SY38♂) (green boxes in Figure 3b). This might be a potential risk of inbreeding.

Three offspring produced from mating between first‐order relatives were found in HZZ (Figure S5) and CDZ (Figure S6). Worse still, all the nine golden snub‐nosed monkeys in HZZ were descendants of a limited number of founders. Most of the individuals in CDZ were descendants of the same pair of parents; thus, most of the juveniles were consanguineous. This increases the risk of inbreeding if they mate with each other after they grow up.

### 3.9 Genetic diversity based on mtDNA D‐loop region sequences

We amplified a total of 157 mtDNA D‐loop region sequences (368 bp in length after being aligned by MEGA) from nine golden snub‐nosed monkey populations and identified 25 haplotypes. In captive populations, haplotype diversity (*h*) ranged from 0.605 (BJW) to 1.0 (BJZ); nucleotide diversity (*π*) ranged from 0.021 (SXB) to 0.069 (BJZ) (Table 7). It indicated that the eight captive populations had relatively high levels of genetic diversity (*h* > 0.5, *π* > 0.005), which was consistent with results based on microsatellite loci. The CDZ had much more haplotypes (11) than other populations. Shared haplotypes were found among different captive populations (Table S9).

**TABLE 7:** Genetic diversity of nine golden snub‐nosed monkey populations based on the mtDNA D‐loop region.

Population	Sample number	Segregating site	Haplotype diversity	Nucleotide diversity
BJZ	3	38	1.000	0.069
BJW	23	39	0.605	0.035
SXB	6	13	0.733	0.021
NJZ	6	32	0.800	0.043
HZZ	9	37	0.750	0.050
SHW	47	41	0.741	0.043
SHZ	16	41	0.892	0.049
CDZ	25	41	0.880	0.047
PW	22	0	0.000	0.000
Total	157	59	0.880	0.046

Unlike captive populations, the haplotype diversity and nucleotide diversity in PW wild population were both zero, indicating no polymorphism in the mitochondrial sequence. Meanwhile, only one singleton (private haplotype) was detected in PW wild population, indicating that individuals in this population may be descendants of a single matrilineal ancestor.

## 4 DISCUSSION

### 4.1 The microsatellite dataset for captive golden snub‐nosed monkeys

As a potential supplement to the wild population, the captive population plays an important role in the conservation of endangered species. However, many previous studies focused on the wild populations of golden snub‐nosed monkeys (Kuang et al., 2019, 2020; Zhao et al., 2016), and very few studies paid attention to the captive populations. Several studies investigated the behavior and disease treatment in zoos (Wang et al., 2021; Yu et al., 2017; Zhao et al., 2016; Zhu et al., 2018), leaving little known about the genetic status and background of captive golden monkeys. Our team conducted a genetic assessment on a captive population of golden snub‐nosed monkeys at Chengdu Zoo (Cai et al., 2020). With the increasing number of captive golden monkeys and captive institutions, most captive populations are very small, with no more than 10 individuals. A national‐wide genetic assessment on the captive populations is of significance and urgently needed for maintaining genetic diversity and keeping healthy development. In this study, we constructed a comprehensive microsatellite dataset for captive golden snub‐nosed monkeys, covering approximately a quarter of the living individuals in China. Based on the dataset, captive institutions can evaluate genetic diversity, understand population structure, design reproduction plans, and identify private alleles of the population. For example, we found several private alleles in BJZ, SHW, SHZ, and CDZ. Individuals with private alleles should be involved in reproduction to preserve the private alleles. However, the current dataset includes genetic information based only on ten microsatellite loci, and it would be helpful to establish a more comprehensive database based on genome‐wide markers such as Single Nucleotide Polymorphisms (SNPs) and include more captive institutions in the future, which will serve as a valuable tool for the unified genetic management of captive populations in the future.

### 4.2 Genetic diversity

We estimated the genetic diversity of eight captive populations and one wild population of golden snub‐nosed monkeys based on microsatellite and mitochondrial markers. The results showed relatively high levels of genetic diversity in the eight captive populations (PIC ranged from 0.43 to 0.542; h ranged from 0.88 to 1; *π* ranged from 0.021 to 0.069). The genetic diversity level in the captive populations is comparable to that in wild populations reported in previous studies (Li et al., 2002; Pan et al., 2005; Wang et al., 2016; Zhang et al., 2019). This may be attributed to the short period of captive breeding programs in China, which is unlikely to impact genetic diversity significantly within two or three decades. The captive institutions usually use individuals from different geographic populations for breeding, which may also result in high levels of genetic diversity. According to the records in the studbook, the captive individuals showed a complex history, which is consistent with the results of Structure analysis and UPGMA clustering. For example, a certain number of rescued wild golden snub‐nosed monkeys have been introduced to CDZ in recent years, providing a genetic supplementation to the captive population. Among the 24 haplotypes identified in the eight captive populations, 11 of which could be found in CDZ. These findings confirmed the complex composition of genetic backgrounds in the captive populations.

Surprisingly, the PW wild population exhibited moderate genetic diversity (PIC = 0.437), lower than all captive populations except BJZ. Meanwhile, only one haplotype was identified in PW, indicating a lack of haplotype diversity and nucleotide diversity. By checking the sample records, we found the PW samples were collected from a small population composing two small one‐male units (OMUs) and one all‐male unit (AMU). Generally, golden snub‐nosed monkeys live in societies characterized by female philopatry, with most females seem to stay in the same OMU, while males transfer between AMUs (Zhang et al., 2006). Given that mitochondrial DNA is maternal inherited, our results suggest that all the PW samples may originate from a single matrilineal clan and there is limited female exchange between this population and other wild populations. It is noteworthy that our sample size of PW population is limited, and there are other wild golden monkey populations in Pingwu forest; our estimation of the genetic diversity cannot represent levels of whole wild populations in Pingwu County.

### 4.3 Genetic structure and differentiation

According to the distribution areas of different subspecies (Groves, 2001; Wang et al., 1998), our genetic clustering indicated that the captive golden snub‐nosed monkeys in the eight zoos mainly constituted two genetic components, which included genetic components from at least two subspecies, *R. r. roxellana* and *R. r. qinlingensis*. However, given that not all individuals in the eight captive institutions are included in the study, individuals from other subspecies may exist in these captive institutions. Compared to simple genetic background in PW wild population, most captive populations had complex genetic composition, especially CDZ and SXB, suggesting significant subspecies hybridization. Artificial crossing of subspecies is a common phenomenon in captive populations for increasing genetic diversity. However, the proposal of subspecies hybridization to genetically rescue endangered species with low genetic diversity in captivity is controversial. Some studies assume hybrid offspring may exhibit a reduction in fitness after releasing into the wild (Christie et al., 2014; Willoughby & Christie, 2019). This is caused by the disruption of advantageous gene complexes, known as outbreeding depression (Frankham et al., 2011). On the contrary, other studies hold an opinion that for inbred populations with low genetic diversity, the conservation of genetic diversity should be considered as priority (Frankham, 2016; Ralls et al., 2018). Given the wide existence of subspecies hybridization in captive golden snub‐nosed monkeys in China, more attention should be paid to monitoring the genetic impacts of subspecies hybridization on the captive populations in the future, especially when the number of hybrid offspring increases quickly.

The analysis of population genetic differentiation (*F*_ST_) revealed a moderate level of genetic differentiation among the eight captive populations (average *F*_ST_ = 0.091). This may be attributed to factors such as genetic drift, inbreeding, and a lack of genetic diversity (Ogden et al., 2020). Further genetic differentiation among captive individuals could lead to reduced fitness when reintroduced in the wild (Willoughby & Christie, 2019).

### 4.4 Parentage analysis and pedigree construction

The parentage identification and genetic pedigree construction in captive populations are important for retaining genetic diversity and preventing inbreeding (Farquharson et al., 2019). Given the small population sizes and the occurrence of inbreeding in captive populations, individuals usually share a close genetic correlation with each other; therefore, highly matched interference is a great challenge to paternity analysis in captive populations. The close relatives of offspring even showed a higher likelihood than the real parents (Thompson, 1976; Thompson & Meagher, 1987). Additionally, other factors such as low DNA quality of fecal samples, genotyping errors, and the presence of null alleles will also cause problems in paternity analysis (Ravinet et al., 2016; Wang, 2011). By carefully eliminating all confounding factors, we used microsatellite‐based paternity test to successfully assign sire and/or dam to 40 offspring of captive golden monkeys and also identified the studbook numbers for eight individuals with unknown identity. However, only 50% of the offspring have biological parents with the highest LOD scores in the single‐blind parental assignment, and about 22% of the genetic parents have the highest LOD scores in double‐blind testing. It may be attributed to the involvement of many closely related siblings in our parentage test. For example, to test the parentage of individual A, if all males in the population are set as candidate fathers, at least two individuals can match A perfectly: A's father and A's son. In addition, 50% of A's siblings are also perfectly matched with A. To get rid of the interference from siblings, we conducted a thorough examination of lineage records, including rearing cage, mating records, and so on. These measures were also suggested by Coetzer et al. (2017) and Ferrie et al. (2013) to improve the accuracy. This enabled us to successfully identify the true biological parents. In fact, when the parentage test is applied in practice, usually only several suspicious candidates are needed for the test, and it is not necessary to test all adult individuals in the population. Therefore, the efficiency of the parentage analysis in practice can be guaranteed.

Although our study indicated high accuracy (35/40, 87.5%) in the manual pedigree records in the eight captive institutions of China, the biological parents of five offspring are inconsistent with the pedigree record parents in our paternity assignments, which was probably due to the incorrect pedigree records. We also found errors in the studbook. For example, according to the studbook of SHW, a pair of parents gave birth to two monkey pups (studbook number 1102 and 1102) within a 3‐day interval, which is obviously impossible in golden snub‐nosed monkeys. Incorrect pedigree records will result in parentage identification and breeding failure in captive institutions and should be avoided by using the genetic pedigree.

### 4.5 Implications to captive population management

Despite the current high levels of genetic diversity in the captive populations, inbreeding occurs in BJZ, HZZ, SHZ, and CDZ. Since we did not sample all individuals in the captive institutions, the inbreeding estimates were just from known portions of the sampled pedigree, and the degree of inbreeding is not known among much of the population as it is not clear that the population traces back to unrelated founders. However, the occurrence of inbreeding found in our study poses a potential threat to inbreeding depression if no strategies are implemented. In addition, subspecies hybridization may result in an unpredictable impact when the captive individuals are introduced to the wild. Therefore, conservation strategies should prioritize the prevention of inbreeding and the maintenance of genetic diversity in captive golden snub‐nosed populations. To achieve this goal, we recommend the following strategies: (1) It is crucial to construct accurate and nationwide genetic pedigrees for all captive individuals and avoid incorrect manual pedigree records; thus, breeding programs based on the genetic pedigree can be developed. (2) To ensure long‐term conservation of genetic diversity, individuals with private and rare alleles should be prioritized in breeding to protect them from disappearing. (3) Genetic exchange between different captive institutions should be promoted, especially between zoos with small population sizes such as HZZ. Only nine individuals live in the HZZ (according to the 2019 studbook), and an inbred offspring (HZZ2) was found in our study. While in SHW with relatively more individuals, many living individuals are the descendants of two alpha males (e.g., 211 and SHW38), which may lead to potential risks of inbreeding. In the future, the development of a kinship matrix, which shows the relatedness among individuals across the entire population, would serve as a valuable tool for identifying breeding pairs with the lowest mean kinship (MK). This approach is considered as the most effective breeding method to maintain genetic diversity and avoid inbreeding in captive populations (Willoughby et al., 2015).

## 5 CONCLUSION

In this study, a genetic assessment for 135 (about a quarter of the whole captive population) captive golden snub‐nosed monkeys was conducted. Although relatively high levels of genetic diversity were estimated in eight captive golden snub‐nosed monkey populations, their genetic structure was quite complicated, indicating the presence of subspecies hybridization. Through paternity identification, the errors in the pedigree records of captive populations were corrected. We further constructed the genetic lineage and found inbred offspring in BJZ, HZZ, SHZ, and CDZ. Therefore, it is recommended to prioritize the prevention of inbreeding when making conservation plans in the future. Our study provides important genetic information on captive golden snub‐nosed monkeys and will be valuable for the genetic management of the captive population in the future.

## CONFLICT OF INTEREST STATEMENT

The authors declare no competing interests.

## Supporting information


Appendix S1.

## Data Availability

All data generated or analyzed during this study are included in this article and Supporting Information.
