# Supplementary material for: Genetic assessment of eight zoo populations of golden snub‐nosed monkey (Rhinopithecus roxellana) implication to the conservation management of captive populations
Source: Evol Appl. 2024 Jun 3;17(6):e13726. doi: 10.1111/eva.13726 (PMC11146145; doi:10.1111/eva.13726)
Supplement: Supplementary file 1 — Appendix S1. [file EVA-17-e13726-s001.docx]

**Table S1 The Captive institutions and population sizes of golden snub-nosed monkey in China (the 2019 International Studbook for Golden Monkey)**

| Location | Size | Location | Size | Location | Size |
| --- | --- | --- | --- | --- | --- |
| **Shanghai Wild Animal Park** | **58** | Harbin Zoological Garden | 8 | Taiyuan Zoological Garden | 4 |
| Tongling Zoo | 51 | Jinan Zoo | 8 | Chongqing Zoo | 4 |
| Dalian Forest Zoological Garden | 48 | **Beijing Zoo** | **7** | Tianjin Zoological Park | 3 |
| Panyu Xiangjiang Safari Park | 41 | Xian Zoological Garden | 7 | Ocean Park Corporation | 3 |
| Jian Zhongnan Baicaoyuan Zoo | 27 | Guangzhou Zoo | 6 | Jinan Wildlife Park | 3 |
| **Shaanxi Rare Wildlife Rescue Base** | **26** | Hefei Wildlife Park | 5 | Ningbo Zoological Garden | 3 |
| **Beijing Wild Animal Park** | **26** | Shangqiu Zoological Garden | 5 | Shipaiwan Country Park | 2 |
| **Nanjing Hongshan Forest Zoo** | **26** | Guiyang Forest Wildlife Park | 5 | Suzhou Zoo | 2 |
| **Shanghai Zoo** | **26** | Fuzhou Zoo | 5 | Xiamen Zhongfeishiye Wildlife Park | 2 |
| Xixiakou Wild Animal Park | 23 | Yantai Zoological Garden | 5 | Nanjing Jinniuhu Wildlife World | 2 |
| **Chengdu Zoo** | **16** | Kunming Zoological Garden | 5 | Wenzhou Zoological Garden | 2 |
| Lanzhou Zoo | 11 | Longsha Wildlife Park | 5 | Nanning Zoological Garden | 2 |
| Hangzhou Wildlife Park | 10 | Zhengzhou Zoo | 4 | Linyi Zoo | 1 |
| Forest Ministry Gansu Endangered Animal Research Center | 9 | Huzhou Longzhimeng Wildlife World | 4 | Fuyang Ecologic Park | 1 |
| **Hangzhou Zoo** | **9** | Shenzhen Safari Park | 4 | Wuhan Zoo | 1 |
| Yangzhou Zoological Garden | 9 | Nanchang Zoological Garden | 4 |  |  |
| Giant Panda Breeding & Research Center | 8 | Shijiazhuang Zoo | 2 | Total | **548** |

*Note*: The study populations in this study were bolded.

**Table S2 Primer information of 10 microsatellite loci**

| Locus | Motif | Primer pair (5 '- 3') | Size (bp) | Location | Chromosome |
| --- | --- | --- | --- | --- | --- |
| GSM03 | CAAA | F: TTCTCCTTCTCTGACACATC  R: TACTGCCAAGTTAGAGTGAG | 154-166 | Non-coding region | 6 |
| GSM04 | CAAA | F: TTGCAGTGAGCCAGGATAGC  R: TTACCTATGAGTGCCAGGCC | 171-187 | Intron | 5 |
| GSM21 | CAAA | F: GCAGGTGGAATGCTTGAACC  R: GCATCTTGCATCTGCTGAGC | 131-155 | Non-coding region | 13 |
| GSM25 | GAAT | F: TGCTTCTAGTGTTTTGCAGTGC  R: GCTGGGCATTAGTGGAGAGG | 129-137 | Non-coding region | 16 |
| GSM32 | ATTT | F: GGTCTGCTTGTTGAATATGGAGC  R: CACACCACTACACTCCAGCC | 168-188 | Intron | 19 |
| GSM42 | ATGA | F: GAAACCAAACTGCCCACACC  R: CACAAAACACCACAGACCGG | 108-128 | Intron | 18 |
| GSM47 | TAGA | F: TCTAGCCTCCAGAACCAT  R: CCTATGTATCTATCTGCCTATC | 155-175 | Intron | 2 |
| GSM51 | CAAA | F: ATCTGGACTGCTTATTCTGT  R: AGGAAGGCTTCATACTCAAG | 120-128 | Non-coding region | 12 |
| GSM69 | TCTT | F: GAGCTAAGTTGTATATTCTGGCTCC  R: CACACCACTGTACTCCAGCC | 134-154 | Non-coding region | 3 |
| GSM75 | TACA | F: AGATGAGATGGTGCCAATG  R: GCCATGCAGGTTGTAGATA | 114-130 | Non-coding region | 4 |

**Table S3 The genotyping and sexing results of 7 captive institutions**

| Sample | GSNM03 | GSNM04 | GSNM21 | GSNM25 | GSNM32 | GSNM42 | GSNM47 | GSNM51 | GSNM69 | GSNM75 | Sex |
| --- | --- | --- | --- | --- | --- | --- | --- | --- | --- | --- | --- |
| Beijing Zoo | | | | | | | | | | | |
| BJZ1 | 166/166 | 175/183 | 131/147 | 137/141 | 168/168 | 120/124 | 155/159 | 134/134 | 138/138 | 126/130 | M |
| BJZ2 | 166/166 | 171/183 | 131/139 | 133/133 | 168/168 | 108/116 | 155/155 | 130/134 | 134/138 | 126/130 | F |
| BJZ3 | 154/154 | 171/171 | 135/135 | 133/133 | 168/168 | 124/128 | 167/171 | 130/134 | 138/138 | 126/126 | M |
| Beijing Wild Animal Park | | | | | | | | | | | |
| BJW1 | 154/166 | 175/183 | 131/131 | 133/141 | 188/188 | 112/124 | 151/163 | 130/134 | 138/138 | 114/126 | M |
| BJW2 | 154/166 | 175/183 | 135/139 | 133/141 | 168/168 | 120/124 | 155/159 | 134/138 | 138/138 | 126/130 | M |
| BJW3 | 154/166 | 171/183 | 131/143 | 133/141 | 168/188 | 108/116 | 155/163 | 134/138 | 138/138 | 114/126 | M |
| BJW4 | 166/170 | 175/175 | 131/147 | 133/141 | 168/188 | 116/124 | 155/155 | 134/138 | 138/138 | 114/126 | F |
| BJW5 | 154/154 | 175/179 | 131/147 | 137/141 | 168/168 | 116/124 | 155/159 | 130/134 | 138/138 | 114/130 | F |
| BJW6 | 154/166 | 175/183 | 139/139 | 133/141 | 168/188 | 116/120 | 155/171 | 134/138 | 138/138 | 126/130 | M |
| BJW7 | 154/166 | 175/179 | 131/139 | 137/141 | 168/168 | 116/124 | 155/159 | 134/138 | 138/138 | 114/130 | F |
| BJW8 | 154/166 | 179/183 | 139/147 | 137/141 | 168/168 | 116/120 | 155/159 | 134/138 | 138/138 | 114/130 | F |
| BJW9 | 154/166 | 179/183 | 139/147 | 137/141 | 168/168 | 116/120 | 155/159 | 134/138 | 138/138 | 114/130 | F |
| BJW10 | 162/166 | 175/183 | 127/131 | 137/141 | 168/192 | 112/124 | 155/167 | 130/134 | 138/138 | 114/126 | F |
| BJW11 | 154/166 | 175/179 | 131/147 | 137/141 | 168/168 | 112/116 | 159/159 | 134/138 | 138/138 | 126/130 | F |
| BJW12 | 166/166 | 171/175 | 131/139 | 137/137 | 168/168 | 112/116 | 155/155 | 130/134 | 138/138 | 114/126 | M |
| BJW13 | 154/166 | 175/179 | 131/147 | 137/141 | 168/168 | 112/116 | 155/155 | 134/138 | 138/138 | 126/130 | F |
| BJW14 | 154/166 | 175/175 | 131/139 | 133/137 | 168/168 | 112/124 | 155/159 | 130/134 | 138/138 | 126/126 | F |
| BJW15 | 154/166 | 175/183 | 131/139 | 133/141 | 168/188 | 112/120 | 155/171 | 130/134 | 138/138 | 114/130 | M |
| BJW16 | 150/154 | 175/183 | 131/139 | 137/137 | 168/188 | 112/116 | 155/163 | 130/134 | 138/138 | 114/130 | M |
| BJW17 | 154/170 | 175/175 | 131/139 | 133/137 | 168/168 | 116/120 | 155/159 | 130/134 | 138/138 | 114/130 | F |
| BJW18 | 162/162 | 179/183 | 127/131 | 141/141 | 168/192 | 116/124 | 155/167 | 130/134 | 138/138 | 122/126 | F |
| BJW19 | 154/166 | 175/179 | 131/139 | 137/141 | 168/168 | 116/120 | 155/155 | 130/134 | 138/138 | 126/130 | M |
| BJW20 | 162/166 | 167/183 | 131/131 | 137/141 | 168/168 | 112/120 | 163/167 | 134/134 | 138/138 | 114/126 | M |
| BJW21 | 154/166 | 175/183 | 135/139 | 133/141 | 168/188 | 116/120 | 155/171 | 130/134 | 138/138 | 114/130 | F |
| BJW22 | 154/166 | 175/183 | 135/139 | 133/133 | 168/188 | 116/116 | 155/159 | 134/138 | 138/138 | 114/126 | F |
| BJW23 | 154/166 | 167/175 | 131/131 | 137/141 | 168/168 | 112/124 | 163/167 | 134/138 | 126/138 | 126/130 | M |
| BJW24 | 154/166 | 175/175 | 131/139 | 133/141 | 168/188 | 112/116 | 159/159 | 130/134 | 130/134 | 122/126 | F |
| Shaanxi Rare Wildlife Rescue Base | | | | | | | | | | | |
| SXB1 | 154/166 | 179/183 | 135/139 | 133/141 | 168/168 | 116/120 | 155/167 | 130/134 | 134/138 | 114/126 | F |
| SXB2 | 166/166 | 179/183 | 131/139 | 137/141 | 168/168 | 116/120 | 155/167 | 130/134 | 130/134 | 114/126 | F |
| SXB3 | 154/166 | 167/183 | 135/139 | 133/141 | 168/168 | 116/120 | 151/163 | 130/134 | 126/134 | 114/126 | M |
| SXB4 | 154/166 | 171/179 | 131/139 | 133/141 | 168/168 | 112/124 | 159/163 | 130/134 | 126/138 | 126/130 | M |
| SXB5 | 154/166 | 171/179 | 135/139 | 129/141 | 168/168 | 116/120 | 151/159 | 134/134 | 134/134 | 114/122 | F |
| SXB6 | 154/166 | 171/179 | 135/139 | 133/137 | 168/168 | 116/120 | 155/159 | 130/134 | 126/138 | 114/134 | M |
| Nanjing Hongshan Forest Zoo | | | | | | | | | | | |
| NJZ1 | 166/166 | 171/171 | 139/143 | 129/133 | 168/188 | 112/116 | 155/159 | 130/134 | 130/134 | 122/126 | F |
| NJZ2 | 166/166 | 171/175 | 135/143 | 133/137 | 168/168 | 108/112 | 159/159 | 130/134 | 134/134 | 126/126 | M |
| NJZ3 | 166/166 | 171/171 | 135/139 | 129/137 | 168/188 | 112/116 | 155/159 | 130/130 | 134/134 | 126/126 | F |
| NJZ4 | 154/166 | 171/183 | 135/139 | 129/133 | 188/188 | 112/116 | 155/155 | 130/130 | 130/134 | 122/126 | F |
| NJZ5 | 154/154 | 183/187 | 127/143 | 133/133 | 168/184 | 112/116 | 155/155 | 130/134 | 134/134 | 114/122 | F |
| NJZ6 | 154/154 | 175/179 | 131/139 | 133/133 | 168/168 | 112/116 | 159/171 | 130/130 | 134/134 | 114/114 | M |
| Hangzhou Zoo | | | | | | | | | | | |
| HZZ1 | 154/166 | 171/183 | 135/139 | 133/141 | 168/168 | 112/116 | 159/175 | 130/130 | 134/134 | 114/122 | F |
| HZZ2 | 166/178 | 175/175 | 135/139 | 129/137 | 168/184 | 112/116 | 155/155 | 130/134 | 134/134 | 114/126 | M |
| HZZ3 | 154/166 | 175/175 | 135/139 | 129/141 | 168/168 | 108/116 | 155/171 | 130/134 | 134/134 | 114/126 | M |
| HZZ4 | 154/166 | 175/175 | 135/139 | 137/141 | 168/168 | 116/116 | 155/155 | 130/134 | 134/134 | 114/122 | F |
| HZZ5 | 166/166 | 175/179 | 135/139 | 129/137 | 168/168 | 112/116 | 155/159 | 130/134 | 134/134 | 126/130 | F |
| HZZ6 | 154/154 | 175/175 | 131/135 | 133/145 | 168/168 | 116/116 | 155/171 | 134/138 | 134/134 | 114/130 | M |
| HZZ7 | 154/162 | 171/183 | 135/139 | 133/145 | 168/168 | 112/116 | 155/155 | 130/134 | 134/134 | 114/130 | F |
| HZZ8 | 162/166 | 175/183 | 135/139 | 137/145 | 168/168 | 116/124 | 155/171 | 134/134 | 130/134 | 114/114 | M |
| HZZ9 | 154/166 | 175/183 | 135/139 | 133/133 | 168/168 | 112/116 | 155/159 | 130/138 | 134/134 | 114/130 | F |
| Shanghai Wild Animal Park | | | | | | | | | | | |
| SHW1 | 154/166 | 171/175 | 135/139 | 133/145 | 168/168 | 112/116 | 155/159 | 134/134 | 134/134 | 114/126 | M |
| SHW2 | 154/154 | 175/175 | 135/139 | 133/133 | 168/168 | 112/116 | 155/155 | 130/134 | 134/134 | 126/126 | M |
| SHW3 | 154/166 | 175/175 | 135/139 | 133/133 | 168/168 | 112/116 | 163/163 | 130/134 | 134/134 | 114/114 | M |
| SHW4 | 154/166 | 171/175 | 135/139 | 133/133 | 168/168 | 112/116 | 159/163 | 130/134 | 134/134 | 126/130 | M |
| SHW5 | 154/166 | 171/175 | 135/139 | 133/141 | 168/168 | 112/116 | 155/159 | 130/130 | 134/134 | 114/126 | F |
| SHW6 | 154/166 | 175/183 | 135/139 | 137/141 | 168/168 | 112/116 | 159/163 | 130/134 | 130/134 | 114/114 | F |
| SHW7 | 154/154 | 175/183 | 135/139 | 133/137 | 168/168 | 112/116 | 155/167 | 134/134 | 134/134 | 114/126 | M |
| SHW8 | 150/150 | 171/175 | 135/139 | 129/133 | 168/168 | 112/116 | 159/167 | 130/134 | 134/134 | 114/130 | M |
| SHW9 | 154/154 | 179/179 | 135/139 | 133/133 | 168/188 | 112/116 | 155/155 | 130/134 | 130/134 | 114/118 | M |
| SHW10 | 154/166 | 167/167 | 135/139 | 129/133 | 160/168 | 112/116 | 159/163 | 130/134 | 130/134 | 126/130 | M |
| SHW11 | 154/166 | 175/175 | 135/139 | 133/133 | 168/168 | 112/116 | 163/163 | 134/134 | 134/134 | 114/114 | M |
| SHW12 | 154/166 | 179/183 | 135/139 | 133/141 | 168/188 | 116/124 | 155/163 | 130/134 | 134/134 | 122/126 | F |
| SHW13 | 166/166 | 175/179 | 135/139 | 133/141 | 168/168 | 116/124 | 155/159 | 130/134 | 130/134 | 122/126 | F |
| SHW14 | 150/162 | 167/171 | 135/139 | 129/141 | 168/188 | 116/120 | 163/167 | 134/138 | 134/134 | 114/126 | F |
| SHW15 | 154/154 | 175/175 | 135/139 | 133/141 | 168/168 | 112/116 | 155/159 | 130/134 | 130/134 | 126/126 | F |
| SHW16 | 154/166 | 183/183 | 135/139 | 133/137 | 168/168 | 112/116 | 163/163 | 130/130 | 130/130 | 122/114 | F |
| SHW17 | 154/166 | 171/175 | 135/139 | 141/137 | 168/168 | 112/116 | 155/163 | 130/134 | 130/134 | 114/126 | F |
| SHW18 | 154/162 | 171/175 | 135/139 | 137/141 | 164/168 | 112/120 | 155/159 | 130/134 | 130/134 | 114/122 | F |
| SHW19 | 154/154 | 171/171 | 135/139 | 133/133 | 168/168 | 116/124 | 155/159 | 130/130 | 134/134 | 114/122 | M |
| SHW20 | 154/154 | 171/171 | 135/139 | 133/137 | 168/188 | 120/128 | 167/175 | 130/134 | 130/134 | 114/126 | F |
| SHW21 | 166/178 | 171/171 | 135/139 | 133/141 | 168/168 | 116/120 | 155/167 | 134/134 | 126/130 | 114/126 | M |
| SHW22 | 154/162 | 175/183 | 135/139 | 133/137 | 168/168 | 116/124 | 155/167 | 130/134 | 134/134 | 114/122 | F |
| SHW23 | 166/166 | 175/179 | 135/139 | 133/141 | 168/168 | 116/124 | 155/167 | 130/134 | 134/134 | 122/126 | M |
| SHW24 | 162/162 | 167/183 | 139/135 | 137/137 | 168/168 | 116/120 | 163/163 | 130/134 | 134/134 | 126/126 | M |
| SHW25 | 166/166 | 175/175 | 135/139 | 137/141 | 168/168 | 112/116 | 155/159 | 130/134 | 134/134 | 114/126 | M |
| SHW26 | 154/166 | 183/171 | 135/139 | 137/141 | 160/188 | 116/124 | 155/159 | 130/134 | 134/134 | 114/126 | M |
| SHW27 | 154/166 | 167/175 | 135/143 | 137/141 | 168/168 | 112/120 | 155/163 | 130/134 | 134/134 | 126/126 | M |
| SHW28 | 154/166 | 175/179 | 135/139 | 137/141 | 168/168 | 112/116 | 155/155 | 130/134 | 134/150 | 114/122 | F |
| SHW29 | 154/166 | 171/179 | 135/139 | 133/141 | 168/168 | 124/124 | 163/167 | 130/134 | 134/134 | 122/126 | F |
| SHW30 | 162/166 | 171/171 | 131/147 | 137/137 | 168/168 | 112/120 | 155/167 | 130/134 | 130/134 | 114/114 | M |
| SHW31 | 154/166 | 175/179 | 135/139 | 133/137 | 168/188 | 112/116 | 159/163 | 130/134 | 134/134 | 114/114 | M |
| SHW32 | 154/166 | 171/179 | 131/135 | 133/145 | 168/168 | 124/128 | 155/167 | 122/134 | 134/134 | 126/126 | M |
| SHW33 | 162/166 | 175/179 | 135/139 | 133/141 | 168/168 | 112/116 | 155/163 | 130/134 | 130/134 | 114/122 | F |
| SHW34 | 154/162 | 171/179 | 135/139 | 133/141 | 168/188 | 120/124 | 155/163 | 130/134 | 130/134 | 114/126 | F |
| SHW35 | 154/166 | 175/183 | 135/139 | 133/137 | 168/168 | 116/116 | 155/159 | 134/138 | 130/134 | 114/114 | F |
| SHW36 | 154/154 | 175/183 | 135/139 | 141/141 | 188/188 | 112/116 | 167/167 | 130/134 | 134/134 | 122/130 | M |
| SHW37 | 162/166 | 171/171 | 135/139 | 137/137 | 168/168 | 112/120 | 155/167 | 130/134 | 130/134 | 114/114 | M |
| SHW38 | 154/162 | 179/183 | 135/139 | 137/141 | 168/188 | 112/116 | 155/163 | 130/134 | 130/134 | 114/122 | M |
| SHW39 | 154/154 | 171/175 | 135/139 | 133/141 | 168/168 | 112/124 | 155/155 | 130/134 | 134/150 | 122/122 | F |
| SHW40 | 154/166 | 179/175 | 135/139 | 133/137 | 168/188 | 112/116 | 155/155 | 130/134 | 134/134 | 122/126 | M |
| SHW41 | 154/166 | 171/179 | 135/139 | 133/141 | 168/168 | 124/124 | 163/167 | 130/134 | 134/134 | 122/126 | F |
| SHW42 | 154/166 | 179/175 | 135/139 | 133/137 | 168/188 | 112/116 | 155/155 | 130/134 | 134/134 | 122/126 | M |
| SHW43 | 154/166 | 179/183 | 135/139 | 133/137 | 160/160 | 112/116 | 159/171 | 134/134 | 134/134 | 114/122 | F |
| SHW44 | 154/166 | 171/171 | 135/139 | 133/141 | 188/188 | 120/124 | 159/159 | 130/134 | 130/134 | 114/126 | F |
| SHW45 | 154/166 | 175/179 | 135/139 | 137/141 | 168/168 | 116/124 | 163/171 | 130/134 | 130/134 | 114/122 | F |
| SHW46 | 154/154 | 171/179 | 135/139 | 133/141 | 184/188 | 112/116 | 163/159 | 130/134 | 130/134 | 118/126 | F |
| SHW47 | 154/166 | 175/179 | 135/139 | 133/141 | 168/184 | 112/124 | 155/163 | 130/134 | 130/134 | 122/126 | F |
| SHW48 | 162/166 | 167/171 | 135/139 | 137/141 | 168/188 | 112/116 | 155/163 | 130/134 | 130/134 | 114/114 | F |
| SHW49 | 154/154 | 171/179 | 135/139 | 133/137 | 168/188 | 112/116 | 155/159 | 130/134 | 130/134 | 114/126 | F |
| Shanghai Zoo | | | | | | | | | | | |
| SHZ1 | 154/166 | 179/183 | 135/139 | 133/141 | 168/168 | 116/124 | 151/151 | 130/134 | 134/134 | 138/138 | F |
| SHZ2 | 154/1 | 171/179 | 135/139 | 133/133 | 168/168 | 112/124 | 155/159 | 134/134 | 134/134 | 126/138 | F |
| SHZ3 | 154/166 | 171/179 | 135/139 | 129/137 | 168/168 | 108/116 | 163/171 | 130/134 | 134/150 | 114/118 | F |
| SHZ4 | 154/154 | 175/183 | 135/139 | 133/133 | 168/168 | 112/116 | 155/159 | 130/134 | 130/134 | 114/130 | F |
| SHZ5 | 154/166 | 175/179 | 135/139 | 133/137 | 168/168 | 112/120 | 155/155 | 130/134 | 134/134 | 114/126 | M |
| SHZ6 | 150/166 | 175/179 | 135/139 | 137/141 | 168/168 | 112/116 | 155/155 | 134/122 | 134/134 | 122/126 | M |
| SHZ7 | 166/166 | 171/175 | 135/139 | 133/137 | 188/188 | 116/124 | 155/159 | 134/130 | 134/134 | 122/126 | F |
| SHZ8 | 166/178 | 171/175 | 135/139 | 137/141 | 168/188 | 116/124 | 155/159 | 130/134 | 134/134 | 114/130 | F |
| SHZ9 | 154/166 | 175/175 | 135/139 | 133/141 | 168/168 | 120/124 | 159/159 | 130/134 | 134/134 | 114/114 | F |
| SHZ10 | 154/154 | 179/179 | 135/139 | 133/141 | 168/188 | 112/112 | 155/159 | 130/134 | 134/150 | 110/114 | F |
| SHZ11 | 154/154 | 171/175 | 135/139 | 133/141 | 168/168 | 108/120 | 155/163 | 134/134 | 130/134 | 114/114 | M |
| SHZ12 | 154/166 | 179/179 | 131/135 | 133/137 | 168/168 | 124/124 | 155/159 | 134/134 | 134/134 | 114/114 | M |
| SHZ13 | 166/166 | 171/171 | 135/139 | 141/141 | 184/188 | 120/124 | 155/155 | 130/134 | 134/134 | 126/130 | M |
| SHZ14 | 154/166 | 175/183 | 135/139 | 133/137 | 188/188 | 116/116 | 155/155 | 130/134 | 134/134 | 126/130 | M |
| SHZ15 | 150/154 | 175/183 | 135/139 | 133/133 | 180/188 | 112/124 | 155/159 | 130/134 | 130/134 | 126/130 | M |
| SHZ16 | 154/166 | 179/179 | 135/131 | 137/137 | 168/168 | 120/124 | 155/155 | 134/134 | 134/134 | 114/130 | F |
| SHZ18 | 154/166 | 175/183 | 135/139 | 133/137 | 188/188 | 116/116 | 155/155 | 130/134 | 134/134 | 126/130 | M |

**Table S4 Genetic diversity of 9 golden snub-nosed monkey populations**

| Locus | Beijing Zoo | | | | | | | | | | | |  | Beijing Wild Animal Park | | | | | | | | |
| --- | --- | --- | --- | --- | --- | --- | --- | --- | --- | --- | --- | --- | --- | --- | --- | --- | --- | --- | --- | --- | --- | --- |
|  | A | | N | H_O_ | | H_E_ | | PIC | *F*_IS_ | | F(null) | |  | A | N | | H_O_ | H_E_ | PIC | | *F*_IS_ | F(null) |
| GSM47 | 4 | | 3 | 0.667 | | 0.8 | | 0.62 | 0.2 | | - | |  | 6 | 23 | | 0.739 | 0.705 | 0.65 | | -0.049 | -0.047 |
| GSM42 | 5 | | 3 | 1 | | 0.933 | | 0.744 | -0.091 | | - | |  | 5 | 23 | | 0.957 | 0.754 | 0.693 | | -0.277 | -0.134 |
| GSM32 | 1 | | 3 | 0 | | 0 | | 0 | - | | - | |  | 3 | 23 | | 0.435 | 0.414 | 0.351 | | -0.053 | -0.05 |
| GSM04 | 3 | | 3 | 0.667 | | 0.733 | | 0.535 | 0.111 | | - | |  | 5 | 23 | | 0.826 | 0.67 | 0.604 | | -0.24 | -0.122 |
| GSM21 | 4 | | 3 | 0.667 | | 0.867 | | 0.671 | 0.273 | | - | |  | 6 | 23 | | 0.826 | 0.696 | 0.631 | | -0.193 | -0.107 |
| GSM25 | 3 | | 3 | 0.333 | | 0.6 | | 0.449 | 0.5 | | - | |  | 3 | 23 | | 0.826 | 0.67 | 0.581 | | -0.24 | -0.127 |
| GSM51 | 2 | | 3 | 0.667 | | 0.533 | | 0.346 | -0.333 | | - | |  | 3 | 23 | | 0.957 | 0.626 | 0.543 | | -0.546 | -0.257 |
| GSM75 | 2 | | 3 | 0.667 | | 0.533 | | 0.346 | -0.333 | | - | |  | 4 | 23 | | 0.957 | 0.704 | 0.626 | | -0.369 | -0.165 |
| GSM69 | 2 | | 3 | 0.333 | | 0.333 | | 0.239 | 0 | | - | |  | 4 | 23 | | 0.087 | 0.128 | 0.122 | | 0.323 | 0.296 |
| GSM03 | 2 | | 3 | 0 | | 0.533 | | 0.346 | 1 | | - | |  | 5 | 23 | | 0.87 | 0.644 | 0.559 | | -0.36 | -0.185 |
| Average | 2.8 | |  | 0.5 | | 0.587 | | 0.43 | 0.178 | | - | |  | 4.4 |  | | 0.748 | 0.601 | 0.536 | | -0.251 | - |
|  |  | |  |  | |  | |  |  | |  | |  |  |  | |  |  |  | |  |  |
| Locus | Shaanxi Rare Wildlife Rescue Base | | | | | | | | | | | |  | Nanjing Hongshan Forest Zoo | | | | | | | | |
|  | A | | N | H_O_ | | H_E_ | | PIC | *F*_IS_ | | F(null) | |  | A | N | | H_O_ | H_E_ | PIC | | *F*_IS_ | F(null) |
| GSM47 | 5 | | 6 | 1 | | 0.864 | | 0.758 | -0.177 | | - | |  | 3 | 6 | | 0.5 | 0.621 | 0.477 | | 0.211 | - |
| GSM42 | 4 | | 6 | 1 | | 0.733 | | 0.596 | -0.429 | | - | |  | 3 | 6 | | 1 | 0.621 | 0.477 | | -0.714 | - |
| GSM32 | 1 | | 6 | 0 | | 0 | | 0 | - | | - | |  | 3 | 6 | | 0.5 | 0.591 | 0.46 | | 0.167 | - |
| GSM04 | 4 | | 6 | 1 | | 0.758 | | 0.639 | -0.364 | | - | |  | 5 | 6 | | 0.667 | 0.742 | 0.643 | | 0.111 | - |
| GSM21 | 3 | | 6 | 1 | | 0.667 | | 0.535 | -0.579 | | - | |  | 5 | 6 | | 1 | 0.818 | 0.708 | | -0.25 | - |
| GSM25 | 4 | | 6 | 1 | | 0.742 | | 0.622 | -0.395 | | - | |  | 3 | 6 | | 0.667 | 0.621 | 0.505 | | -0.081 | - |
| GSM51 | 2 | | 6 | 0.833 | | 0.53 | | 0.368 | -0.667 | | - | |  | 2 | 6 | | 0.5 | 0.409 | 0.305 | | -0.25 | - |
| GSM75 | 5 | | 6 | 1 | | 0.758 | | 0.644 | -0.364 | | - | |  | 3 | 6 | | 0.5 | 0.682 | 0.555 | | 0.286 | - |
| GSM69 | 4 | | 6 | 0.833 | | 0.758 | | 0.639 | -0.111 | | - | |  | 2 | 6 | | 0.333 | 0.303 | 0.239 | | -0.111 | - |
| GSM03 | 2 | | 6 | 0.833 | | 0.53 | | 0.368 | -0.667 | | - | |  | 2 | 6 | | 0.167 | 0.53 | 0.368 | | 0.706 | - |
| Average | 3.4 | | 6 | 0.85 | | 0.634 | | 0.517 | -0.389 | | - | |  | 3.1 | 6 | | 0.583 | 0.594 | 0.474 | | 0.012 | - |
|  |  | |  |  | |  | |  |  | |  | |  |  |  | |  |  |  | |  |  |
| Locus | Hangzhou Zoo | | | | | | | | | | | |  | Shanghai Wild Animal Park | | | | | | | | |
|  | A | | N | H_O_ | | H_E_ | | PIC | *F*_IS_ | | F(null) | |  | A | N | | H_O_ | H_E_ | PIC | | *F*_IS_ | F(null) |
| GSM47 | 4 | | 9 | 0.667 | | 0.601 | | 0.522 | -0.116 | | - | |  | 6 | 47 | | 0.646 | 0.77 | 0.725 | | -0.031 | -0.024 |
| GSM42 | 4 | | 9 | 0.778 | | 0.575 | | 0.48 | -0.383 | | - | |  | 5 | 47 | | 0.958 | 0.713 | 0.654 | | -0.354 | -0.17 |
| GSM32 | 2 | | 9 | 0.111 | | 0.111 | | 0.099 | 0 | | - | |  | 5 | 47 | | 0.271 | 0.446 | 0.399 | | 0.209 | 0.123 |
| GSM04 | 4 | | 9 | 0.556 | | 0.595 | | 0.512 | 0.07 | | - | |  | 5 | 47 | | 0.625 | 0.758 | 0.708 | | 0.077 | 0.033 |
| GSM21 | 3 | | 9 | 1 | | 0.582 | | 0.448 | -0.8 | | - | |  | 5 | 47 | | 1 | 0.546 | 0.434 | | -0.846 | -0.309 |
| GSM25 | 5 | | 9 | 0.778 | | 0.824 | | 0.742 | -0.067 | | - | |  | 5 | 47 | | 0.604 | 0.689 | 0.618 | | -0.134 | -0.069 |
| GSM51 | 3 | | 9 | 0.778 | | 0.627 | | 0.505 | -0.258 | | - | |  | 4 | 47 | | 0.875 | 0.542 | 0.431 | | -0.564 | -0.23 |
| GSM75 | 4 | | 9 | 0.889 | | 0.699 | | 0.611 | -0.293 | | - | |  | 5 | 47 | | 0.708 | 0.708 | 0.649 | | -0.022 | -0.014 |
| GSM69 | 2 | | 9 | 0.111 | | 0.111 | | 0.099 | 0 | | - | |  | 4 | 47 | | 0.479 | 0.444 | 0.38 | | -0.165 | -0.08 |
| GSM03 | 4 | | 9 | 0.778 | | 0.673 | | 0.565 | -0.167 | | - | |  | 5 | 47 | | 0.583 | 0.672 | 0.605 | | -0.083 | -0.055 |
| Average | 3.5 | | 9 | 0.634 | | 0.54 | | 0.458 | -0.228 | | - | |  | 4.9 | 47 | | 0.723 | 0.613 | 0.542 | | -0.183 |  |
|  |  | |  |  | |  | |  |  | |  | |  |  |  | |  |  |  | |  |  |
| Locus | Shanghai Zoo | | | | | | | | | | | |  | Chengdu Zoo | | | | | | | | |
|  | A | | N | H_O_ | | H_E_ | | PIC | *F*_IS_ | | F(null) | |  | A | N | | H_O_ | H_E_ | PIC | | *F*_IS_ | F(null) |
| GSM47 | 5 | | 16 | 0.563 | | 0.615 | | 0.539 | 0.088 | | 0.012 | |  | 6 | 25 | | 0.56 | 0.803 | 0.758 | | 0.307 | 0.153 |
| GSM42 | 5 | | 16 | 0.813 | | 0.788 | | 0.724 | -0.032 | | -0.032 | |  | 6 | 25 | | 0.56 | 0.794 | 0.746 | | 0.299 | 0.159 |
| GSM32 | 4 | | 16 | 0.25 | | 0.478 | | 0.402 | 0.485 | | 0.345 | |  | 3 | 25 | | 0.48 | 0.545 | 0.428 | | 0.122 | 0.053 |
| GSM04 | 4 | | 16 | 0.688 | | 0.744 | | 0.669 | 0.078 | | 0.026 | |  | 5 | 25 | | 0.6 | 0.666 | 0.596 | | 0.101 | 0.054 |
| GSM21 | 3 | | 16 | 1 | | 0.573 | | 0.456 | -0.791 | | -0.303 | |  | 5 | 25 | | 0.68 | 0.744 | 0.684 | | 0.088 | 0.045 |
| GSM25 | 4 | | 16 | 0.688 | | 0.688 | | 0.601 | 0 | | -0.015 | |  | 4 | 25 | | 0.28 | 0.468 | 0.408 | | 0.406 | 0.265 |
| GSM51 | 3 | | 16 | 0.75 | | 0.506 | | 0.397 | -0.506 | | -0.221 | |  | 3 | 25 | | 0.56 | 0.582 | 0.475 | | 0.039 | 0.012 |
| GSM75 | 7 | | 16 | 0.75 | | 0.786 | | 0.728 | 0.048 | | 0.009 | |  | 5 | 25 | | 0.72 | 0.716 | 0.643 | | -0.006 | -0.013 |
| GSM69 | 3 | | 16 | 0.313 | | 0.284 | | 0.257 | -0.103 | | -0.078 | |  | 3 | 25 | | 0.28 | 0.398 | 0.353 | | 0.302 | 0.179 |
| GSM03 | 4 | | 16 | 0.688 | | 0.603 | | 0.496 | -0.146 | | -0.085 | |  | 4 | 25 | | 0.28 | 0.313 | 0.28 | | 0.109 | 0.034 |
| Average | 4.2 | | 16 | 0.65 | | 0.607 | | 0.527 | -0.074 | |  | |  | 4.4 | 25 | | 0.5 | 0.603 | 0.537 | | 0.174 |  |
|  |  | |  |  | |  | |  |  | |  | |  |  |  | |  |  |  | |  |  |
| Locus | | Pingwu wild population | | | | | | | | | | | | | | | | | |  |  |  |
|  |  | A | | | N | | H_O_ | | | H_E_ | | PIC | | | | *F*_IS_ | | F(null) | |  |  |  |
| GSM47 | | 3 | | | 22 | | 0.818 | | | 0.627 | | 0.557 | | | | -0.319 | | -0.163 | |  |  |  |
| GSM42 | | 5 | | | 22 | | 0.5 | | | 0.481 | | 0.436 | | | | -0.041 | | -0.013 | |  |  |  |
| GSM32 | | 4 | | | 22 | | 0.591 | | | 0.464 | | 0.403 | | | | -0.281 | | -0.159 | |  |  |  |
| GSM04 | | 3 | | | 22 | | 0.818 | | | 0.635 | | 0.549 | | | | -0.297 | | -0.135 | |  |  |  |
| GSM21 | | 3 | | | 22 | | 0.318 | | | 0.321 | | 0.292 | | | | 0.01 | | 0.024 | |  |  |  |
| GSM25 | | 3 | | | 22 | | 0.727 | | | 0.667 | | 0.579 | | | | -0.093 | | -0.06 | |  |  |  |
| GSM51 | | 2 | | | 22 | | 0.773 | | | 0.585 | | 0.5 | | | | -0.332 | | -0.179 | |  |  |  |
| GSM75 | | 3 | | | 22 | | 0.136 | | | 0.212 | | 0.197 | | | | 0.364 | | 0.2 | |  |  |  |
| GSM69 | | 3 | | | 22 | | 0.455 | | | 0.495 | | 0.367 | | | | 0.083 | | 0.031 | |  |  |  |
| GSM03 | | 3 | | | 22 | | 0.455 | | | 0.571 | | 0.49 | | | | 0.208 | | 0.135 | |  |  |  |
| Average | | 3.1 | | | 22 | | 0.559 | | | 0.506 | | 0.437 | | | | -0.109 | |  | |  |  |  |

*Note*: Allele number at the locus (A), number of individuals typed at the locus(N), observed heterozygosity (H_O_), expected heterozygosity (H_E_), polymorphic information content (PIC), inbreeding coefficient (*F*_IS_).

**Table S5 Genetic diversity of 9 golden snub-nosed monkey populations**

| Locus | A | N | H_O_ | H_E_ | PIC |
| --- | --- | --- | --- | --- | --- |
| GSM47 | 7 | 157 | 0.707 | 0.752 | 0.71 |
| GSM42 | 6 | 157 | 0.809 | 0.736 | 0.697 |
| GSM32 | 7 | 157 | 0.369 | 0.534 | 0.481 |
| GSM04 | 6 | 157 | 0.72 | 0.758 | 0.713 |
| GSM21 | 7 | 157 | 0.822 | 0.723 | 0.669 |
| GSM25 | 5 | 157 | 0.694 | 0.68 | 0.62 |
| GSM51 | 4 | 157 | 0.771 | 0.568 | 0.476 |
| GSM75 | 8 | 157 | 0.682 | 0.703 | 0.651 |
| GSM69 | 6 | 157 | 0.357 | 0.606 | 0.534 |
| GSM03 | 7 | 157 | 0.592 | 0.589 | 0.511 |
| Average | 6.3 | 157 | 0.652 | 0.665 | 0.606 |

*Note*: Allele number at the locus (A), number of individuals typed at the locus(N), observed heterozygosity (H_O_), expected heterozygosity (H_E_), polymorphic information content (PIC)

**Table S6 The origin of the 135 samples used in this study according to the studbook record. Wild-caught individuals were shown in bold, and the regions of individuals born in zoos indicate the regions of their parents/grandparents according to the studbook**

| Sample name | Studbook number | Name | Origin |
| --- | --- | --- | --- |
| BJZ1 | 392 | DAZHUANG | (GANSU & SHAANXI) & (SICHUAN & SHAANXI) |
| BJZ2 | 957 | PEIQI | SHAANXI |
| BJZ3 | 940 | QIUQIU | SHAANXI |
| BJW1 | 935 | NIANNIAN | (SICHUAN &SHAANXI) & (SICHUAN & GANSU) |
| BJW2 | 762 | DALI | SICHUAN & (SICHUAN & SHAANXI) |
| BJW3 | 375 | XIAOYUE | (SHAANXI & GANSU) & SICHUAN |
| BJW4 | 550 | QIFU | (SICHUAN & GANSU) & SHAANXI |
| BJW5 | 439 | XIAOHUA | (SHAANXI & GANSU) & (SHAANXI & SICHUAN) |
| BJW6 | 416 | YIXIONG | SICHUAN |
| BJW7 | 1034 | PANPAN | SICHUAN & (SICHUAN & SHAANXI) |
| BJW9 | 277 | XIAOER | SICHUAN & SHAANXI |
| BJW10 | NULL | XIAOBAO | UNKNOW |
| BJW11 | 813 | SIXI | (GANSU & SHAANXI & SICHUAN) & (GANSU & SHAANXI & SICHUAN) |
| BJW12 | 1055 | XIAOMING | (GANSU & SHAANXI) & (GANSU & SHAANXI & SICHUAN) |
| BJW13 | 805 | LAIYA | (GANSU & SHAANXI) & (SICHUAN & SHAANXI) |
| BJW14 | 589 | XIAOHEI | SICHUAN & GANSU |
| BJW15 | 272 | XIAOMEI | SICHUAN |
| BJW16 | 823 | DUODUO | GANSU & (GANSU & SICHUAN) |
| BJW17 | 857 | SANYUE | SICHUAN & GANSU |
| BJW18 | 319 | DANIU | UNKNOW |
| BJW19 | 342 | SANNIU | UNKNOW |
| BJW20 | 337 | LIUMEI | SICHUAN |
| BJW21 | 295 | SIMEI | SICHUAN & SHAANXI |
| BJW22 | 512 | DONGDONG | GANSU & SICHUAN |
| BJW23 | 504 | XIAOJIAN | (SHAANXI & GANSU) &SICHUAN |
| BJW24 | 1100 | LIUTIAN | (SHAANXI & SICHUAN) & SICHUAN |
| SXB1 | 486 | YUNYUN | TAIBAI (SHAANXI) & SHAAANXI |
| SXB2 | 1013 | LILI | TAIBAI (SHAANXI) & SHAAANXI |
| SXB3 | NULL | SONGSONG | UNKNOW |
| SXB4 | 477 | JUNJUN | TAIBAI (SHAANXI) |
| SXB5 | 496 | YINGYING | TAIBAI & (TAIBAI & SHAANXI) |
| **SXB6** | 481 | HUANGHUANG | SHAANXI |
| NJZ1 | NULL | JINXIAOMENG | (WUDU (GANSU) & SHAANXI) & (GANSU & SHAANXI) |
| NJZ2 | 442 | XIAOWEI | GANSU & SHAANXI |
| NJZ3 | 775 | SISI | WUDU (GANSU) & SHAANXI |
| NJZ4 | 1085 | TONGCHENG | WUDU (GANSU) & SHAANXI |
| NJZ5 | 838 | ERWAN | WUDU (GANSU) & SHAANXI |
| NJZ6 | 691 | CHONGYANG | GANSU & SHAANXI |
| SHW1 | 584 | 117 | GANSU & (SHAANXI & SICHUAN) |
| SHW2 | 674 | 123 | GANSU & SHAANXI |
| SHW3 | 419 | 73 | GANSU & SHAANXI |
| **SHW4** | 615 | T1 | SHAANXI |
| SHW5 | NULL | 202 | UNKNOW & SHAANXI |
| SHW6 | 852 | 162 | SHAANXI & GANSU |
| SHW7 | 973 | 171 | (SHAANXI & GANSU) & SHAANXI |
| SHW8 | 676 | 121 | (SHAANXI & GANSU) & GANSU |
| SHW9 | UNKNOWN |  |  |
| SHW10 | 1047 | 191 | UNKNOW |
| SHW11 | 591 | 113 | (SHAANXI & GANSU) & GANSU |
| SHW12 | 1058 | 192 | GANSU & SHAANXI |
| SHW13 | 1074 | 194 | UNKNOW |
| SHW14 | 217 | 982 | GANSU |
| SHW15 | 993 | 182 | GANSU & (SHAANXI & GANSU) |
| SHW16 | 542 | 102 | GANSU & SHAANXI |
| SHW17 | 449 | 82 | SHAANXI & GANSU |
| SHW18 | 661 | 122 | GANSU & SHAANXI |
| SHW19 | NULL | 201 | UNKNOWN & SHAANXI |
| SHW20 | 931 | 176 | UNKNOWN |
| SHW21 | 540 | 101 | GANSU & SHAANXI |
| SHW22 | 955 | 1710 | GANSU & SHAANXI |
| SHW23 | 708 | 133 | GANSU & SHAANXI |
| SHW24 | 446 | 83 | GANSU & SHAANXI |
| SHW25 | 747 | 141 | GANSU & SHAANXI |
| SHW26 | 444 | 81 | GANSU & SHAANXI |
| SHW27 | 579 | 115 | GANSU & SHAANXI |
| SHW28 | NULL | 2010 | GANSU & SHAANXI |
| SHW29 | 485 | T4 | SHAANXI |
| SHW30 | 892 | TUANTUAN | TAIBAI (SHAANXI) & SHAAANXI |
| SHW31 | 841 | 155 | GANSU & SHAANXI |
| SHW32 | 752 | 145 | GANSU & SHAANXI |
| SHW33 | 1098 | 198 | GANSU & SHAANXI |
| SHW34 | 1082 | 196 | GANSU & SHAANXI |
| SHW35 | 616 | T2 | SHAANXI |
| SHW36 | 821 | 153 | GANSU & SHAANXI |
| SHW37 | 928 | 173 | UNKNOWN |
| SHW38 | 420 | 75 | GANSU & SHAANXI |
| SHW39 | 490 | T5 | SHAANXI |
| SHW40 | 1047 | 193 | SHAANXI |
| SHW43 | 1098 | 1910 | GANSU & SHAANXI |
| SHW44 | UNKNOWN |  |  |
| SHW45 | NULL | 208 | GANSU & SHAANXI |
| SHW46 | 479 | T3 | UNKNOWN |
| SHW47 | 677 | 124 | GANSU |
| SHW48 | 926 | 174 | (GANSU & SHAANXI ) & SHAANXI |
| SHW49 | 932 | 178 | GANSU & SHAANXI |
| SHZ1 | 214 | BEIBEI | GANSU & (SICHUAN & UNKNOWN) |
| SHZ2 | 244 | QINGQING | GANSU & SHAANXI |
| SHZ3 | 744 | ERNIU | GANSU |
| SHZ4 | 787 | YUEYUE | GANSU & SHAANXI |
| SHZ5 | 698 | HAOHAO | GANSU |
| SHZ6 | 790 | LIANGLIANG | GANSU & (GANSU & SICHUAN & UNKNOWN) |
| SHZ7 | 963 | ANJI | SHAANXI & (SHAANXI & UNKNOWN) |
| SHZ8 | 570 | MAOMAO | GANSU & SHAANXI |
| SHZ9 | 568 | QINQIN | GANSU & (SICHUAN & SHAANXI & UNKNOWN) |
| SHZ10 | 891 | XIAOXIA | GANSU |
| SHZ11 | 902 | MEIZAI | GANSU & SHAANXI |
| SHZ12 | 835 | ANZAI | GANSU |
| SHZ13 | 829 | NULL | GANSU & (GANSU & SHAANXI) |
| SHZ15 | 888 | NULL | GANSU & SHAANXI |
| SHZ16 | 1095 | ANNA | GANSU |
| SHZ18 | 759 | XIAOXI | GANSU |
| HZZ1 | 869 | ZEPING | SHAANXI & (SHAANXI & GANSU) |
| HZZ2 | NULL | YASHENG | GANSU & SHAANXI |
| HZZ3 | 349 | LUOKE | GANSU & SHAANXI |
| HZZ4 | 853 | YAAN | GANSU |
| HZZ5 | 990 | YAQIN | GANSU |
| HZZ6 | NULL | HUQIANG | GANSU & (SHAANXI & GANSU) |
| HZZ7 | 1025 | HUHANG | GANSU & (SHAANXI & GANSU) |
| HZZ8 | 543 | YAHU | GANSU |
| HZZ9 | 441 | LINGLING | GANSU & SHAANXI |
| CDZ1 | 920 | NULL | (GANSU & SICHUAN & UNKNOWN) & SHAANXI |
| CDZ2 | 667 | YUYU | UNKNOWN & SHAANXI |
| CDZ3 | 868 | LEILEI | SICHUAN & (SICHUAN & GANSU & UNKNOWN) |
| CDZ4 | 428 | DUDU | SICHUAN & (SICHUAN & GANSU & UNKNOWN) |
| CDZ5 | 741 | JIAJIA | SICHUAN & (SICHUAN & GANSU & UNKNOWN) |
| CDZ6 | 966 | LONGZI | GANSU & SICHUAN |
| CDZ7 | 429 | QINGQING | GANSU & (SICHUAN & UNKNOWN) |
| CDZ8 | 391 | CHUNCHUN | GANSU & SICHUAN |
| CDZ9 | 898 | TIAOTIAO | GANSU & (SICHUAN & UNKNOWN) |
| CDZ10 | 757 | ZIZI | GANSU & (SICHUAN & UNKNOWN) |
| CDZ11 | 593 | TUTU | SHAANXI |
| CDZ12 | 414 | FAFA | SICHUAN |
| CDZ13 | 599 | XIXI |  |
| CDZ14 | UNKNOWN |  |  |
| CDZ15 | UNKNOWN |  |  |
| **CDZ16** | 779 | BOBO | SICHUAN |
| CDZ17 | 997 | XIAOXIAO | SICHUAN & (SICHUAN & GANSU & UNKNOWN) |
| CDZ18 | UNKNOWN |  | SICHUAN & (SICHUAN & GANSU & UNKNOWN) |
| CDZ19 | NULL | HANHAN |  |
| CDZ20 | 402 | CHENGCHENG | SICHUAN & (SICHUAN & GANSU & UNKNOWN) |
| CDZ21 | NULL | HUAHUA |  |
| **CDZ22** | 283 | LONGLONG | SICHUAN |
| CDZ23 | UNKNOWN |  |  |
| **CDZ24** | 629 | HONGHONG | SICHUAN |
| CDZ25 | UNKNOWN |  |  |

**Table S7 Single-blind paternity testing. Pedigree records were underlined.**

| Offspring ID | Father ID/Mother ID | Number of analyzed loci | Pair Loci Mismatched | LOD | Δ | Confidence | Note |
| --- | --- | --- | --- | --- | --- | --- | --- |
| BJZ1 ♂ | BJW9 ♀ | 10 | 0 | 1.79 | 1.21 | >95% |  |
|  | Other 10 candidates | 10 | 0 ~ 3 | -11.72 ~ 0.58 |  |  |  |
| BJW1 ♂ | BJW4 ♀ | 10 | 1 | -2.91 |  |  |  |
|  | Other 10 candidates | 10 | 2 ~ 4 | -16.83 ~ -6.03 |  |  |  |
| BJW4 ♀ | BJW17 ♀ | 10 | 0 | 0.51 | 0.4 |  | Not reached child-bearing age /kinship |
|  | Other 6 candidates | 10 | 0 ~ 1 | -4.52 ~ 0.12 |  |  |  |
|  | BJW9 ♀ | 10 | 1 | -4.54 |  |  |  |
| BJW5 ♀ | BJW11 ♀ | 10 | 0 | 2.07 | 0.12 |  | Not reached child-bearing age |
|  | BJW9 ♀ | 10 | 0 | 1.95 |  |  |  |
|  | Other 2 candidates | 10 | 0 | 0.90 ~ 1.39 |  |  |  |
| BJW11 ♀ | BJW1 ♂ | 10 | 1 | -1.77 |  |  | Physical separation |
|  | BJW23 ♂ | 10 | 1 | -3.07 |  |  |  |
|  | Other 4 candidates | 10 | 1~2 | -9.23~-5.56 |  |  |  |
| BJW12 ♂ | BJW14 ♀ | 10 | 0 | 2.16 | 2.04 | >95% |  |
|  | Other 10 candidates | 10 | 0 ~ 3 | -13.00 ~ 0.11 |  |  |  |
| BJW13 ♀ | BJW9 ♀ | 10 | 0 | 2.04 | 0.65 | >80% |  |
|  | Other 10 candidates | 10 | 0 ~ 2 | -8.14 ~ 1.39 |  |  |  |
| BJW14 ♀ | BJW13 ♀ | 10 | 0 | -0.53 |  |  | Not reached child-bearing age |
|  | BJW19 ♀ | 10 | 0 | -0.72 |  |  |  |
|  | Other 8 candidates | 10 | 1~3 | -12.58~-3.1 |  |  |  |
| BJW16 ♂ | BJW17 ♀ | 10 | 0 | 0.02 | 0.02 | >80% | Not reached child-bearing age |
|  | Other 3 candidates | 10 | 0 ~ 1 | -3.91 ~ --1.67 |  |  |  |
|  | BJW22 ♀ | 10 | 1 | -4.74 |  |  |  |
|  | Other 4 candidates | 10 | 1~3 | -14.1~-4.81 |  |  |  |
| BJW17 ♀ | BJW19 ♀ | 10 | 0 | 1.08 | 0.997 | >80% |  |
|  | Other 12 candidates | 10 | 0~4 | -17.2~0.91 |  |  |  |
| BJW20 ♀ | BJW18 ♀ | 10 | 1 | -1.7 |  |  | Not reached child-bearing age |
|  | BJW15 ♀ | 10 | 1 | -4.42 |  |  |  |
|  | Other 10 candidates | 10 | 1~4 | -16.32~-2.86 |  |  |  |
| BJW23 ♂ | BJW11 ♀ | 10 | 0 | -3.07 |  |  | Not reached child-bearing age |
|  | Other 4 candidates | 10 | 1 | -5.42 ~ -3.07 |  |  |  |
|  | BJW18 ♀ | 10 | 2 | -8.45 |  |  |  |
|  | Other 4 candidates | 10 | 2~4 | -17.03~-9.52 |  |  |  |
| BJW24 ♂ | BJW11 ♀ | 10 | 1 | -4.21 |  |  | Not reached child-bearing age |
|  | BJW17 ♀ | 10 | 2 | -7.04 |  |  |  |
|  | BJW19 ♀ | 10 | 2 | -8.56 |  |  |  |
|  | Other 8 candidates | 10 | 2~4 | -15.1~-8.9 |  |  |  |
| NJZ4 ♀ | NJZ2 ♂ | 10 | 2 | -11 |  |  |  |
|  | NJZ6 ♂ | 10 | 4 | -16 |  |  |  |
| NJZ5 ♀ | NJZ6 ♂ | 10 | 3 | -9.95 |  |  |  |
|  | NJZ2 ♂ | 10 | 4 | -16.2 |  |  | Wrong pedigree record |
| HZZ1 ♀ | HZZ9 ♀ | 10 | 0 | 1.15 | 1.15 | >95% |  |
| HZZ2 ♂ | HZZ3 ♂ | 10 | 0 | -0.44 |  |  |  |
| SHW6 ♀ | SHW16 ♀ | 10 | 0 | 3.8 | 1.41 | >80% | Not reached child-bearing age |
|  | SHW35 ♀ | 10 | 0 | 2.39 |  |  |  |
|  | SHW17 ♀ | 10 | 0 | 1.63 |  |  |  |
|  | Other 10 candidates | 10 | 0 ~ 3 | -11.7 ~ 0.32 |  |  |  |
| SHW8 ♂ | SHW14 ♀ | 10 | 0 | 3.28 | 3.28 | >95% |  |
|  | Other 13 candidates | 10 | 1 ~ 4 | -15.9 ~ -4.79 |  |  |  |
| SHW19 ♂ | SHW4 ♂ | 10 | 1 | -1.83 |  |  |  |
|  | Other 16 candidates | 10 | 1 ~ 5 | -19.3 ~ -2.72 |  |  |  |
| SHW48 ♀ | SHW26♂ | 10 | 0 | -0.41 |  |  | Kinship |
|  | SHW38♂ | 10 | 1 | -0.61 |  |  |  |
|  | SHW21 ♂ | 10 | 0 | -0.9 |  |  |  |
|  | Other 14 candidates | 10 | 1 ~ 4 | -15.8 ~ -0.97 |  |  |  |
| SHZ13 ♂ | SHZ8 ♀ | 10 | 1 | -2.33 |  |  |  |
|  | SHZ9 ♀ | 10 | 4 | -13.7 |  |  |  |

**Table S8 Double-blind paternity testing. Pedigree records were underlined**

| Offspring ID | Parent combination （Mother & Father） | Number of analyzed loci | Trio Loci Mismatched | Trio LOD | Δ | Trio Confidence | Note |
| --- | --- | --- | --- | --- | --- | --- | --- |
| BJW2 ♂ | BJW22♀ & BJW6♂ | 10 | 1 | 0.43 | 0.43 | >80% | Kinship |
|  | BJW5♀ & BJW6♂ | 10 | 1 | -1.82 |  |  |  |
| BJW7 ♀ | BJW9♀ & BJZ1♂ | 10 | 0 | 4.76 |  |  | Kinship |
|  | BJW8♀ & BJZ1♂ | 10 | 0 | 4.76 |  |  |  |
|  | BJW5♀ & BJW2♂ | 10 | 0 | 3.41 |  |  |  |
|  | BJW5♀ & BJW6♂ | 10 | 0 | 2.73 |  |  |  |
|  | Other 4 combinations | 10 | 1 | 0.05~0.70 |  |  |  |
| NJZ1 ♀ | NJZ2♀ & NJZ3♂ | 10 | 2 | -5.15 |  |  |  |
|  | Other 5 combinations | 10 | 6 | -23.20 ~-21.12 |  |  |  |
| HZZ6 ♂ | HZZ9♀ & HZZ8♂ | 10 | 2 | -4.67 |  |  |  |
| HZZ7 ♀ | HZZ9♀ & HZZ8♂ | 10 | 1 | -0.73 |  |  |  |
| SHW5 ♀ | SHW17♀ & SHW4♂ | 10 | 0 | 4.01 | 1.37 | >95% | Physical separation |
|  | SHW17♀ & SHW31♂ | 10 | 0 | 2.64 |  |  |  |
|  | SHW18♀ & SHW4♂ | 10 | 0 | 1.97 |  |  |  |
|  | Other 9 combinations | 10 | 0 ~ 1 | 0.03 ~ 1.65 |  |  |  |
| SHW10 ♂ | SHW46♀ & SHW8 ♂ | 10 | 3 | -6.11 |  |  |  |
|  | Other 6 combinations | 10 | 3 ~ 5 | -20 ~ -6.53 |  |  |  |
|  | SHW47♀ & SHW38 ♂ | 10 | 5 | -20.2 |  |  | Wrong pedigree record |
| SHW12 ♀ | SHW29♀ & SHW26 ♂ | 10 | 0 | 5.96 | 1.35 | >95% | Physical separation |
|  | SHW29♀ & SHW38 ♂ | 10 | 0 | 4.61 |  |  |  |
|  | Other 4 combinations | 10 | 0 ~ 1 | 0.90 ~ 3.92 |  |  |  |
| SHW13 ♀ | SHW47♀ & SHW25 ♂ | 10 | 0 | 5.15 | 0.7 | >95% |  |
|  | SHW47♀ & SHW04 ♂ | 10 | 0 | 4.45 |  |  |  |
|  | Other 179 combinations | 10 | 0 ~ 5 | -17 ~ 3.78 |  |  |  |
|  | SHW16♀ & SHW3 ♂ | 10 | 5 | -17 |  |  | Wrong pedigree record |
| SHW15 ♀ | SHW17♀ & SHW4 ♂ | 10 | 0 | 4.6 | 0.68 | >80% | Physical separation |
|  | SHW17♀ & SHW1 ♂ | 10 | 0 | 3.92 |  |  |  |
|  | Other 4 combinations | 10 | 0 ~ 1 | 1.25 ~ 3.24 |  |  |  |
| SHW20 ♀ | SHW44♀ & SHW32 ♂ | 10 | 2 | -0.39 |  |  |  |
|  | Other 97 combinations | 10 | 2 ~ 5 | -16.5 ~ -2.39 |  |  |  |
|  | SHW35♀ & SHW38 ♂ | 10 | 4 | -16.7 |  |  | Wrong pedigree record |
| SHW22 ♀ | SHW35♀ & SHW23 ♂ | 10 | 1 | 0.37 | 0.09 | >80% | Physical separation |
|  | SHW39♀ & SHW38 ♂ | 10 | 1 | 0.28 |  |  |  |
|  | SHW35♀ & SHW38 ♂ | 10 | 1 | 0.11 |  |  |  |
| SHW28 ♀ | SHW25♀ & SHW39 ♂ | 10 | 1 | 3.9 | 1.08 | >95% | Physical separation |
|  | Other 9 combinations | 10 | 1 ~ 2 | -1.53 ~ 2.82 |  |  |  |
|  | SHW35♀ & SHW38 ♂ | 10 | 1 | -1.53 |  |  |  |
| SHW31 ♂ | SHW43♀ & SHW3 ♂ | 10 | 1 | 2.57 | 0.014 | >80% | Not reached child-bearing age |
|  | SHW35♀ & SHW38 ♂ | 10 | 0 | 2.56 |  |  |  |
|  | Other 3 combinations | 10 | 1 | 0.93 ~ 1.94 |  |  |  |
| SHW33 ♀ | SHW35♀ & SHW38 ♂ | 10 | 0 | 3.57 | 0.66 | >80% |  |
|  | Other 9 combinations | 10 | 0 ~ 1 | 0.53 ~ 2.91 |  |  |  |
| SHW34 ♀ | SHW14♀ & SHW09 ♂ | 10 | 0 | 1.75 | 1.75 | >95% | Physical separation |
|  | SHW29♀ & SHW38 ♂ | 10 | 1 | -0.04 |  |  | Already pregnant |
|  | SHW46♀ & SHW38 ♂ | 10 | 1 | -1.47 |  |  |  |
| SHW37 ♂ | SHW18♀ & SHW21 ♂ | 10 | 1 | 1.56 | 1.56 | >95% |  |
|  | Other 118 combinations | 10 | 2 ~ 5 | -2.34 ~ -18.6 |  |  |  |
|  | SHW47♀ & SHW38 ♂ | 10 | 5 | -18.7 |  |  | Wrong pedigree record |
| SHW49 ♀ | SHW9♀ & SHW26 ♂ | 10 | 0 | 4.58 | 0.71 | >80% | Physical separation |
|  | SHW17♀ & SHW31 ♂ | 10 | 0 | 3.88 |  |  |  |
|  | SHW46♀ & SHW38 ♂ | 10 | 0 | 3.21 |  |  | SHW46 to be 479 |
|  | Other 8 combinations | 10 | 0 ~ 1 | 0.02 ~ 1.82 |  |  |  |

**Table S9 Distribution of haplotypes in mtDNA D-loop region of *Rhinopithecus roxellana***

| Haplotype | Population | | | | | | | | | Total |
| --- | --- | --- | --- | --- | --- | --- | --- | --- | --- | --- |
|  | BJZ | BJW | SXB | NJZ | HZZ | SHW | SHZ | CDZ | PW |  |
| Hap_1 | 1 | 14 | 3 | 0 | 1 | 16 | 3 | 0 | 0 | 38 |
| Hap_2 | 1 | 0 | 0 | 0 | 0 | 0 | 0 | 7 | 0 | 8 |
| Hap_3 | 1 | 0 | 0 | 0 | 0 | 0 | 0 | 5 | 0 | 6 |
| Hap_4 | 0 | 1 | 0 | 0 | 0 | 0 | 0 | 0 | 0 | 1 |
| Hap_5 | 0 | 3 | 0 | 0 | 0 | 0 | 0 | 0 | 0 | 3 |
| Hap_6 | 0 | 4 | 0 | 0 | 3 | 0 | 2 | 0 | 0 | 9 |
| Hap_7 | 0 | 1 | 0 | 0 | 0 | 0 | 0 | 0 | 0 | 1 |
| Hap_8 | 0 | 0 | 2 | 1 | 0 | 0 | 0 | 0 | 0 | 3 |
| Hap_9 | 0 | 0 | 1 | 0 | 0 | 0 | 0 | 0 | 0 | 1 |
| Hap_10 | 0 | 0 | 0 | 0 | 1 | 13 | 3 | 3 | 0 | 20 |
| Hap_11 | 0 | 0 | 0 | 0 | 0 | 0 | 0 | 1 | 0 | 1 |
| Hap_12 | 0 | 0 | 0 | 0 | 0 | 0 | 0 | 1 | 0 | 1 |
| Hap_13 | 0 | 0 | 0 | 0 | 0 | 0 | 0 | 1 | 0 | 1 |
| Hap_14 | 0 | 0 | 0 | 0 | 4 | 13 | 3 | 2 | 0 | 22 |
| Hap_15 | 0 | 0 | 0 | 1 | 0 | 4 | 0 | 2 | 0 | 7 |
| Hap_16 | 0 | 0 | 0 | 0 | 0 | 0 | 0 | 1 | 0 | 1 |
| Hap_17 | 0 | 0 | 0 | 0 | 0 | 0 | 0 | 1 | 0 | 1 |
| Hap_18 | 0 | 0 | 0 | 0 | 0 | 0 | 0 | 1 | 0 | 1 |
| Hap_19 | 0 | 0 | 0 | 0 | 0 | 0 | 0 | 0 | 22 | 22 |
| Hap_20 | 0 | 0 | 0 | 3 | 0 | 0 | 0 | 0 | 0 | 3 |
| Hap_21 | 0 | 0 | 0 | 1 | 0 | 0 | 0 | 0 | 0 | 1 |
| Hap_22 | 0 | 0 | 0 | 0 | 0 | 0 | 3 | 0 | 0 | 3 |
| Hap_23 | 0 | 0 | 0 | 0 | 0 | 0 | 1 | 0 | 0 | 1 |
| Hap_24 | 0 | 0 | 0 | 0 | 0 | 1 | 1 | 0 | 0 | 2 |
| Hap_25 | 0 | 0 | 0 | 0 | 0 | 1 | 0 | 0 | 0 | 1 |
| Total | 3 | 5 | 3 | 4 | 4 | 6 | 7 | 11 | 1 | 25 |


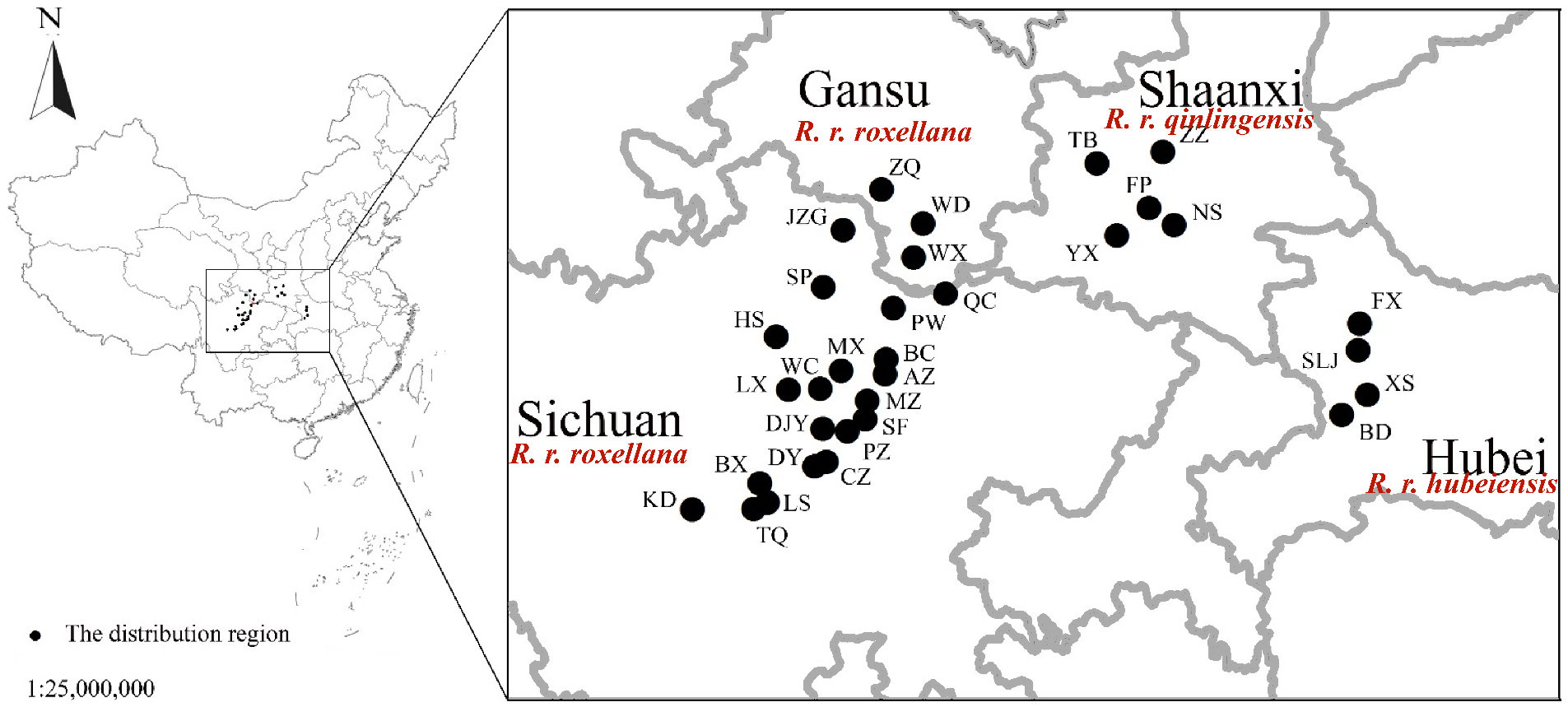


**Figure S1 Distribution map of golden snub-nosed monkeys (Yao et al., 2022)**


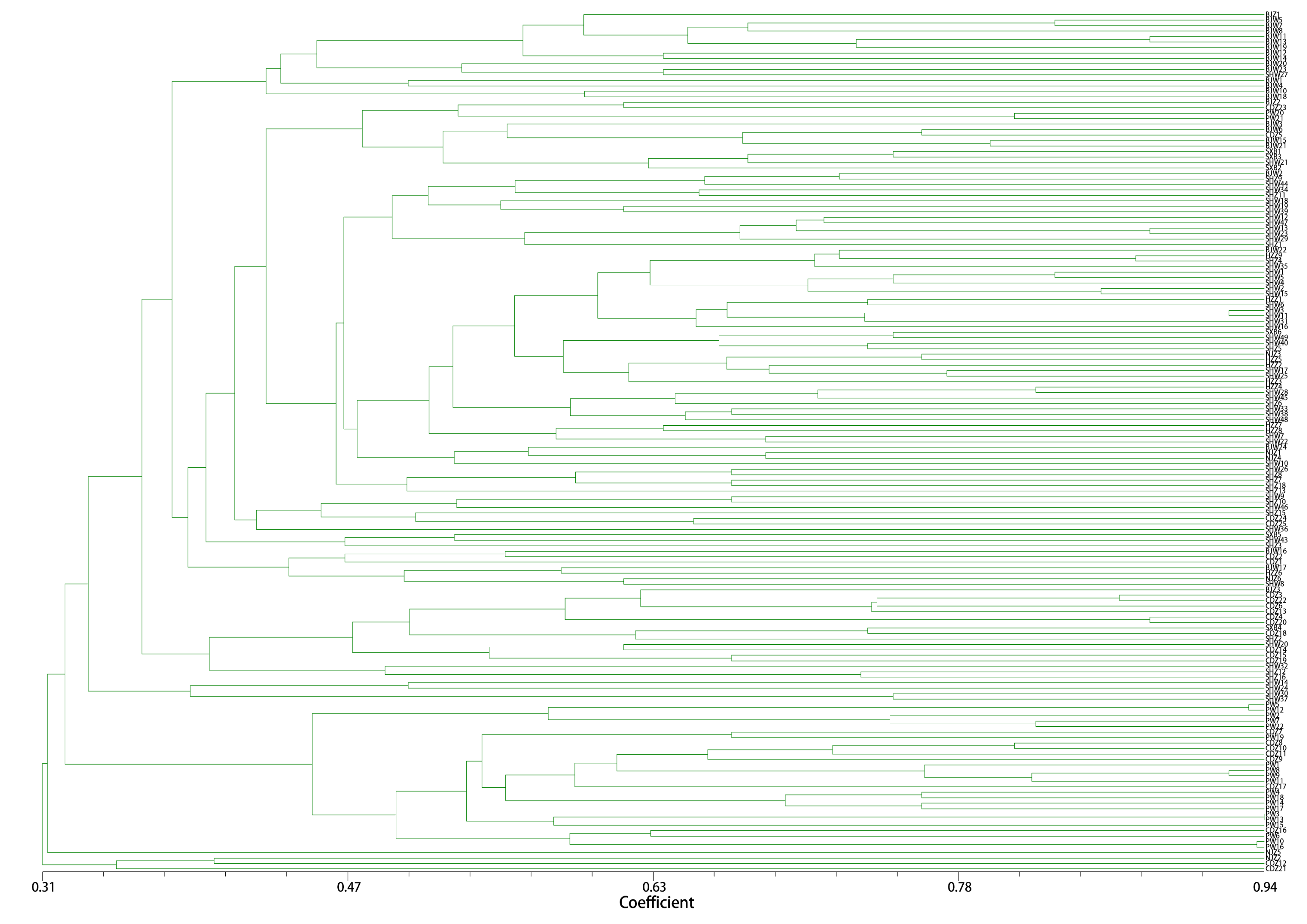


**Figure S2 Dendrogram of 157 golden snub-nosed monkeys generated using UPGMA based on microsatellite genotypes.**


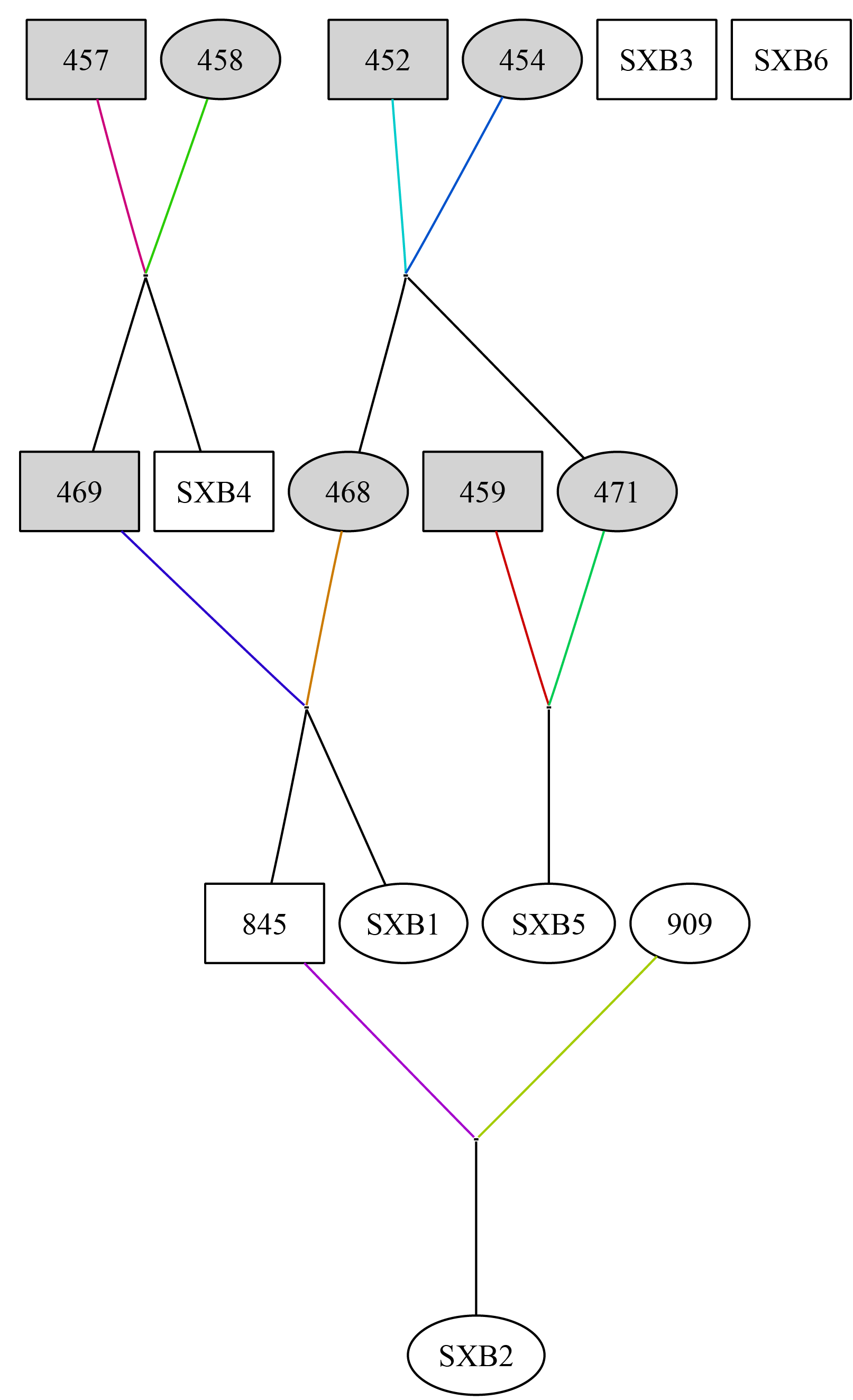


**Figure S3 Genetic pedigree of Shaanxi Rare Wildlife Rescue Base (SXB). 🞏: male, ⭘: female,** **dead individuals were filled in grey. Various colored lines represent different mating combinations of parents. When two colored lines intersect, if the black solid line leads down from the intersection point, it represents the following individuals as their offspring.**


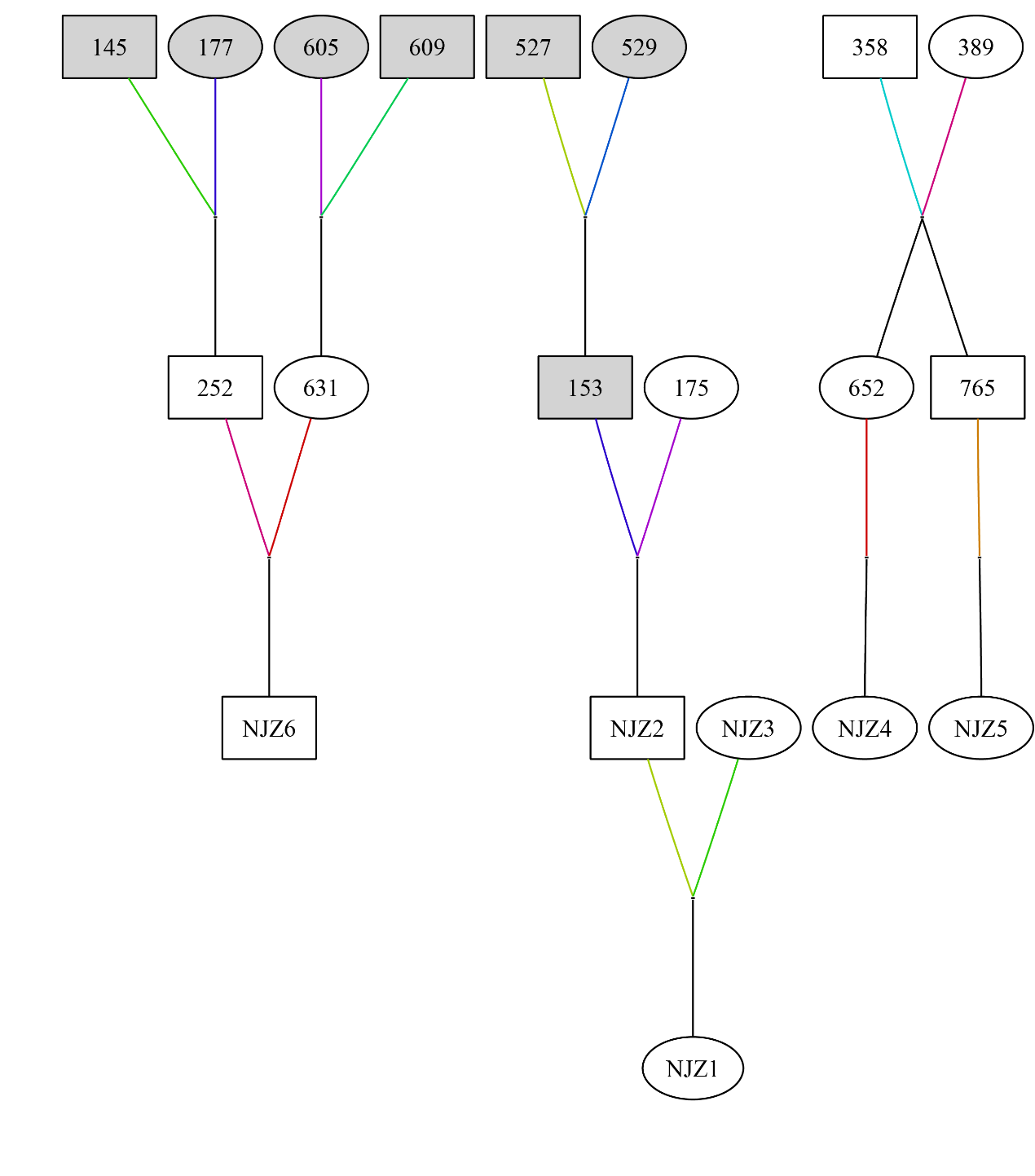


**Figure S4 Genetic pedigree of Nanjing Hongshan Forest Zoo (NJZ). 🞏: male, ⭘: female, dead individuals were filled in grey. Various colored lines represent different mating combinations of parents. When two colored lines intersect, if the black solid line leads down from the intersection point, it represents the following individuals as their offspring.**


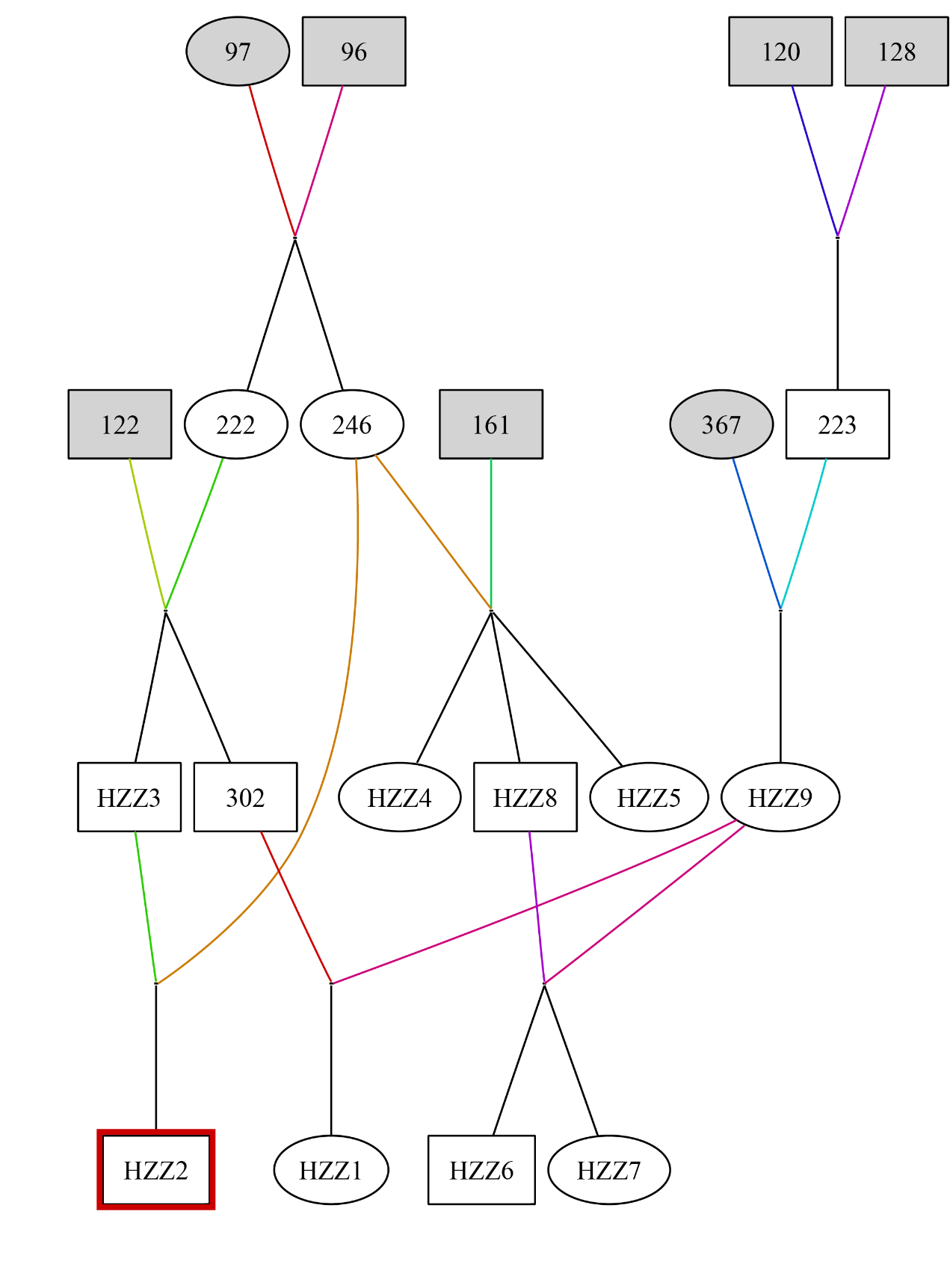


**Figure S5 Genetic pedigree of Hangzhou Zoo (HZZ). 🞏: male, ⭘: female, dead individuals were filled in grey; inbreeding individuals were marked in red. Various colored lines represent different mating combinations of parents. When two colored lines intersect, if the black solid line leads down from the intersection point, it represents the following individuals as their offspring.**


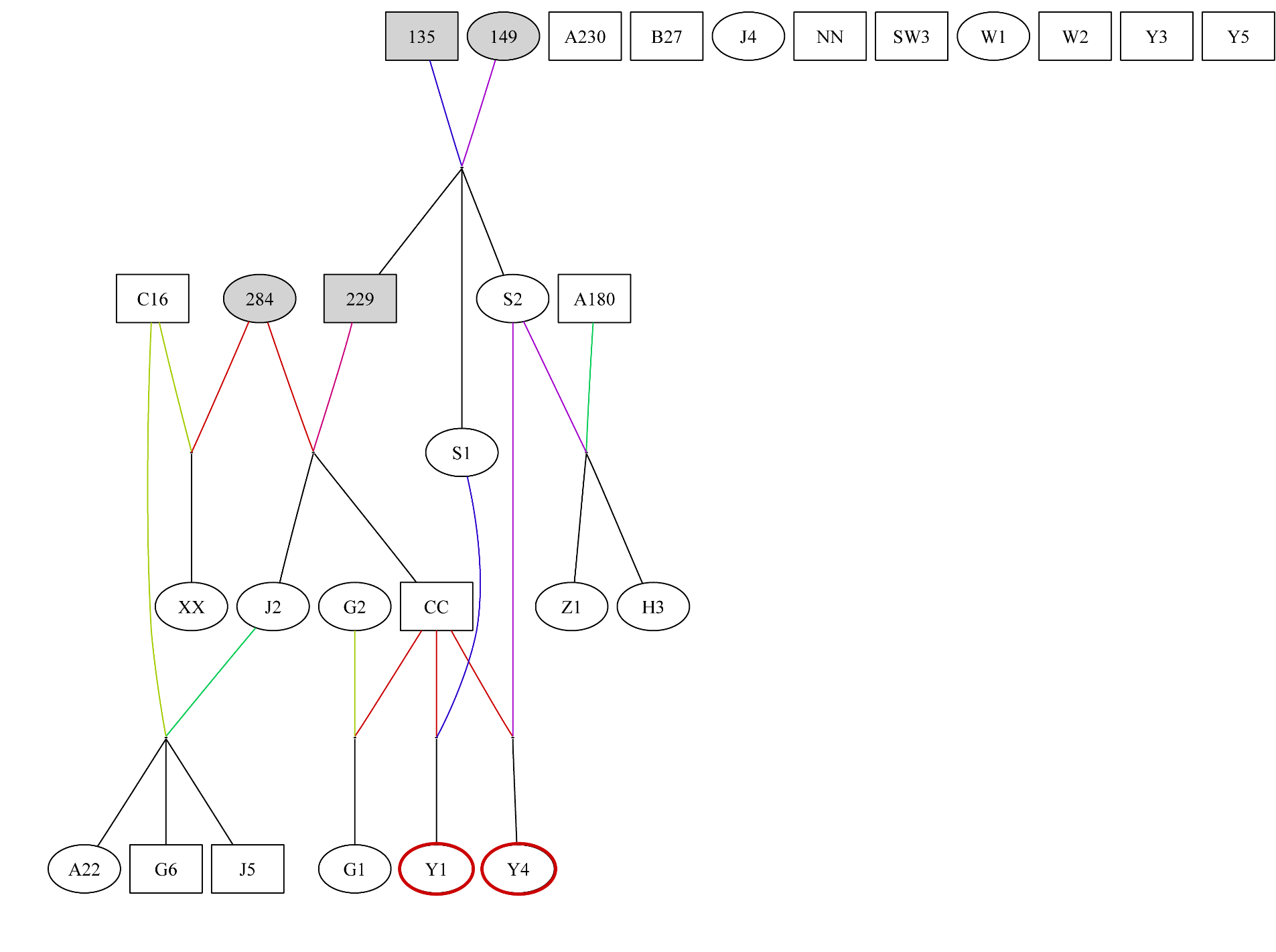


**Figure S6 Genetic pedigree of Chengdu Zoo (CDZ). 🞏: male, ⭘: female, dead individuals were filled in grey; inbreeding individuals were marked in red. Various colored lines represent different mating combinations of parents. When two colored lines intersect, if the black solid line leads down from the intersection point, it represents the following individuals as their offspring.**

# Reference

Yao, G., Fan, Y., Li, D., Hull, V., Shen, L., Li, Y., & Hu, J. (2022). The Influence of Environmental Variables on Home Range Size and Use in the Golden Snub-Nosed Monkey (*Rhinopithecus roxellana*) in Tangjiahe National Nature Reserve, China. *Animals : an open access journal from MDPI, 12*(18), 2338. doi: https://doi.org/10.3390/ani12182338
